# Cardioprotecive Properties of Known Agents in Rat Ischemia-Reperfusion Model Under Clinically Relevant Conditions: Only the NAD Precursor Nicotinamide Riboside Reduces Infarct Size in Presence of Fentanyl, Midazolam and Cangrelor, but Not Propofol

**DOI:** 10.3389/fcvm.2021.712478

**Published:** 2021-08-30

**Authors:** Yang Xiao, Philippa Phelp, Qian Wang, Diane Bakker, Rianne Nederlof, Markus W. Hollmann, Coert J. Zuurbier

**Affiliations:** ^1^Laboratory of Experimental Intensive Care and Anesthesiology, Department of Anesthesiology, Amsterdam Cardiovascular Sciences, Amsterdam University Medical Centres, University of Amsterdam, Amsterdam, Netherlands; ^2^Institut für Herz- und Kreislaufphysiologie, Heinrich- Heine- Universität Düsseldorf, Düsseldorf, Germany

**Keywords:** cardioprotection, animal model, infarct size, NAD+ precursor, P2Y_12_ antagonist, propofol, benzodiazepine, fentanyl

## Abstract

**Background:** Cardioprotective strategies against ischemia-reperfusion injury (IRI) that remain effective in the clinical arena need to be developed. Therefore, maintained efficacy of cardioprotective strategies in the presence of drugs routinely used clinically (e.g., opiates, benzodiazepines, P2Y_12_ antagonist, propofol) need to be identified in preclinical models.

**Methods:** Here, we examined the efficacy of promising cardioprotective compounds [fingolimod (Fingo), empagliflozin (Empa), melatonin (Mela) and nicotinamide riboside (NR)] administered i.v. as bolus before start ischemia. Infarct size as percentage of the area of risk (IS%) was determined following 25 min of left ascending coronary (LAD) ischemia and 2 h of reperfusion in a fentanyl-midazolam anesthetized IRI rat model. Plasma lactate dehydrogenase (LDH) activity at 30 min reperfusion was determined as secondary outcome parameter. Following pilot dose-response experiments of each compound (3 dosages, *n* = 4–6 animals per dosage), potential cardioprotective drugs at the optimal observed dosage were subsequently tested alone or in combination (*n* = 6–8 animals per group). The effective treatment was subsequently tested in the presence of a P2Y_12_ antagonist (cangrelor; *n* = 6/7) or propofol aesthesia (*n* = 6 both groups).

**Results:** Pilot studies suggested potential cardioprotective effects for 50 mg/kg NR (*p* = 0.005) and 500 μg/kg melatonin (*p* = 0.12), but not for Empa or Fingo. Protection was subsequently tested in a new series of experiments for solvents, NR, Mela and NR+Mela. Results demonstrated that only singular NR was able to reduce IS% (30 ± 14 vs. 60 ± 16%, *P* = 0.009 vs. control). Mela (63 ± 18%) and NR+Mela (47 ± 15%) were unable to significantly decrease IS%. NR still reduced IS in the presence of cangrelor (51 ± 18 vs. 71 ± 4%, *P* = 0.016 vs. control), but lost protection in the presence of propofol anesthesia (62 ± 16 vs. 60 ± 14%, *P* = 0.839 vs. control). LDH activity measurements supported all IS% results.

**Conclusion:** This observational study suggests that NR is a promising cardioprotective agent to target cardiac ischemia-reperfusion injury in clinical conditions employing opioid agonists, benzodiazepines and platelet P2Y_12_ inhibitors, but not propofol.

## Background

Since the seminal discovery in 1986 of the intrinsic potential of the heart to protect itself against acute cardiac ischemia-reperfusion injury (IRI) (1), there has been an enormous gain in knowledge concerning the underlying mechanisms and protection of acute cardiac IRI (2–4). The current challenge is to translate the preclinical knowledge for successful clinical application to combat cardiac IRI in, for instance, cardiac surgery, percutaneous coronary interventions (PCI), or cardiogenic shock. Thus far, translation has been disappointing, which is due, at least partly, to the mismatch between preclinical and clinical conditions. One important mismatch concerns the preclinical lack of co-medications and comorbidities often present in patients (5). In addition, despite many parallel molecular mechanisms that contribute to IRI in the preclinical setting, mostly singular target therapy has been examined in the clinical scenario. Monotherapy contrasts the knowledge that IRI constitutes a multifactorial process (6). Activating multiple survival pathways within one treatment is likely necessary to achieve protection in a heterogenous population of humans subjected to IRI, as compared to the rather homogenous laboratory animals employed in preclinical models. Improved matching of preclinical experimental conditions to clinical reality and the development of multitarget IRI therapy are now recognized as critical new steps to harness the preclinical knowledge into effective clinical treatment (6).

In the current study we explored several of these new steps in our *in vivo* model of cardiac IRI. Firstly, we have incorporated fentanyl (opioid) and midazolam (benzodiazepine) as core components of our standard preclinical anesthetic regimen. Opioids and benzodiazepines are often administered during PCI and were reported to affect IRI or its protection thereof (7–10), necessitating the testing of potential cardioprotective interventions in the presence of these anesthetic and analgesic agents. Secondly, we examined four previously reported cardioprotective compounds for efficacy with fentanyl-midazolam anesthesia, with as goal to find at least two effective compounds used in a multitarget therapy. The compounds tested were: (1) fingolimod, a spingosine-1 agonist, showing cardioprotection in several models (11, 12), (2) empagliflozin, a sodium glucose cotransporter 2 (SGLT2) inhibitor, a novel clinical diabetes and heart failure medicine class, showing promise as cardioprotective agent for cardiac IRI (13), (3) melatonin, an antioxidant, demonstrating protection in various models of cardiac IRI (14, 15), and (4) nicotinamide riboside (NR), a precursor of the reducing compound NAD^+^, known to be depleted during ischemia (16). Although several studies have shown that nicotinamide mononucleotide (NMN), another precursor of NAD^+^, or NAD^+^ itself, can offer cardioprotection against cardiac IRI (17–19), such information is currently missing for NR. NR has been suggested to be ideal for boosting tissue NAD^+^ due to its fast tissue penetration and raising tissue NAD^+^. Most of these four compounds have shown protection against cardiac IRI. However, protection has not been tested in the presence of a fentanyl-midazolam anesthetic regimen. Following pilot studies examining dose-response curves for these compounds, the most promising agents were subsequently tested alone or in combination. Finally, the most effective therapy was tested in the presence of a platelet antagonist because recent studies have demonstrated that these agents, nowadays almost always present during PCI, often nullified preclinical cardioprotective interventions (20, 21). The most protective strategy was also tested with total intravenous anesthesia (TIVA) employing propofol because TIVA is often used during cardiac surgery and propofol is known to interfere with various cardioprotective interventions (22, 23). Additionally, because hyperglycemia is often prevented during clinical cardiac ischemia-reperfusion conditions due to the reported high glucose contribution to ischemic damage and poor outcome, we also monitored blood glucose levels with our different interventions, knowing that background anesthesia may have pronounced effects on glucose homeostasis (24, 25). Importantly, the major goal of this work is to evaluate efficacy of several established cardioprotective agents on a much more clinically relevant background therapy than is commonly performed in preclinical animal models. Therefore, elucidation of possible underlying mechanisms of successful or failed protection was not performed. We believe that to improve preclinical to clinical translation, research is needed that focus specifically and solely on what is working under clinical conditions. This is in line with current recommendations to improve successful drug discovery for cardiovascular disease, with emphasis on the development of models more relevant to human diseases and conditions (26). Here, we hypothesized that one or two of the established compounds may still provide cardioprotection in the presence of fentanyl-midazolam anesthesia, cangrelor and/or propofol anesthesia.

## Methods

### Animals

This study was approved by the Animal Ethics Committee of the Academic Medical Center, Amsterdam, The Netherlands (approved DEC protocol # DAA62). All procedures were performed in accordance with the Guide for the Use and Care of Laboratory Animals and the guidelines from Directive 2010/63/EU of the European Parliament on the protection of animals used for scientific purposes. Adult Wistar rats (330 ± 31 grams, Charles River, Lyon, France) were used in different experimental protocols. Male rats were used to minimize hormonal effects. For acclimatization, rats were housed for at least 1 week in standard housing conditions (12 h dark/12 h light cycle with food and water *ad libitum*) in the Animal Research Institute AMC (ARIA) before use.

### Cardiac I/R *in vivo*

Surgical preparation was performed as reported previously (23). Briefly, rats were anesthetized intraperitoneally (4.25 ml/kg) with a mixture of FMA [Fentanyl (0.15 mg/kg), Midazolam (7.5 mg/kg) and Acepromazine (2.5 mg/kg)]. Rats were positioned on a heating pad with a heating lamp to maintain body temperature (37°C) which was monitored by a rectal probe. Rats were then intubated and mechanically ventilated (30% oxygen) at a respiratory rate of 60 breaths/min and an inspiratory pressure of 10 mbar. The tail vein was cannulated to supply maintenance anesthesia at 2.5 ml/kg/h (92 μg/kg/h fentanyl, 1.85 mg/kg/h midazolam, and 2.8 mg/kg/h acepromazine), the left or right carotid artery was cannulated for recoding aortic pressure and the corresponding jugular vein was cannulated for fluid maintenance (10 ml/kg/h, with 0.84% bicarbonate in saline) and drug infusion. For assessment of electrocardiograms (ECG), bipolar ECG leads were used, for which needles were inserted subcutaneously under the right forepaw and below the left rib cage. A lateral left-sided thoracotomy in the fourth costal space was performed, and a ligature (5–0 Prolene) was passed under the left anterior descending (LAD) coronary artery. The ends of the suture were threaded through a small piece of tubing to create a snare that could be clamped to occlude the LAD. Aortic pressure and ECG were digitized using an analog-to-digital converter (PowerLab 16/35, ADInstruments Pty Ltd, Castle Hill, Australia) and Bio Amp (FE231, ADInstruments), respectively, and were continuously recorded using LabChart Pro 8 software (ADInstruments).

### Experimental Protocol *in vivo*

**Figure 1:**
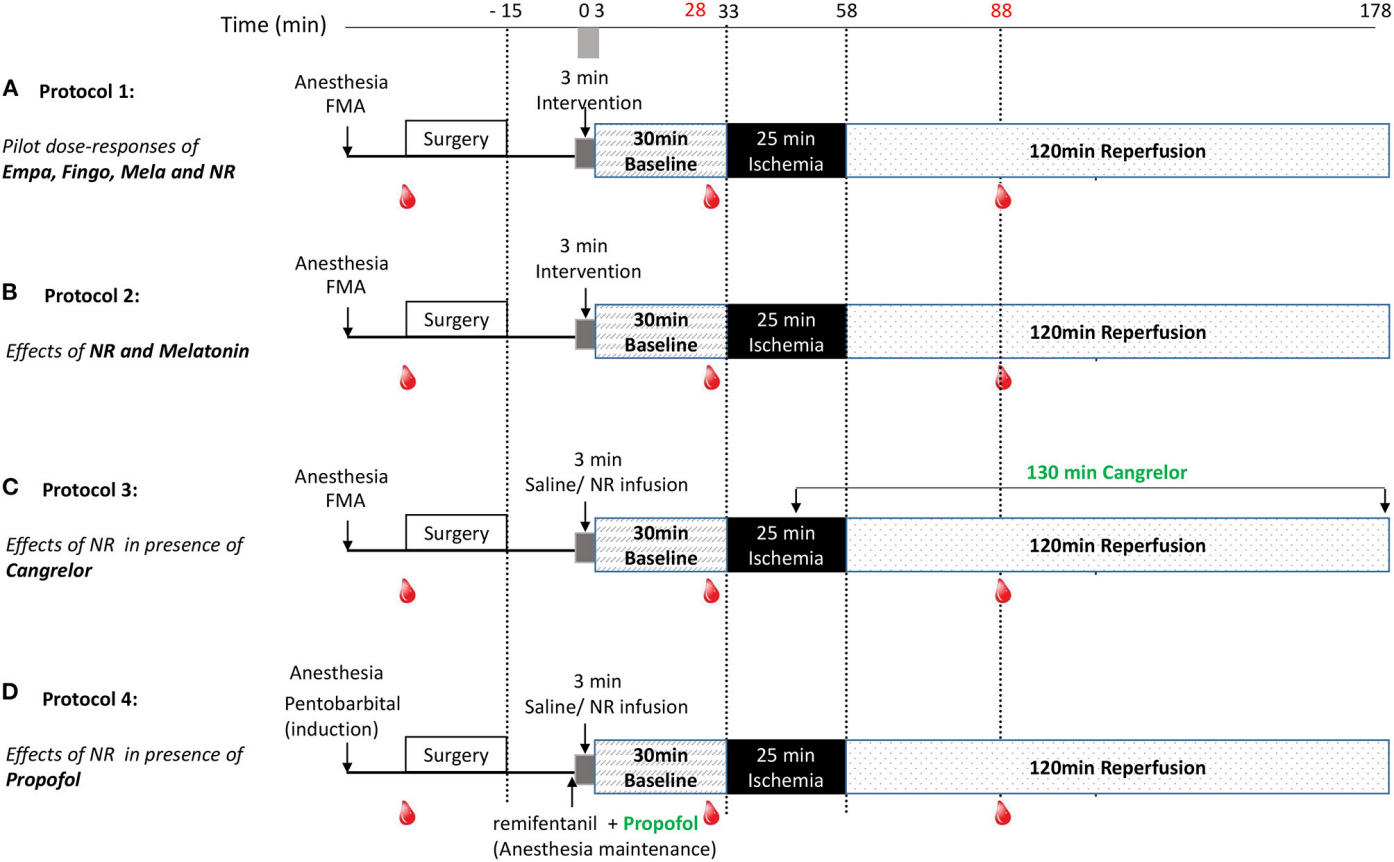
Experimental protocols. Following surgery and 3 min drug infusion, rat hearts were subjected to 30 min stabilization, 25 min ischemia (I) and 120 min reperfusion (R). Blood samples were collected before surgery (0.15 ml), 5 min before ischemia (0.15 ml) and 30 min after reperfusion (0.15 + 1 ml); **(A)** Protocol 1: For examining dose-responses of Empagliflozin, Fingolimod, nicotinamide riboside (NR) and Melatonin, rats were randomly assigned to 12 groups: saline, 10, 25, or 125 mg/kg empagliflozin, 1, 3, or 10 mg/kg Fingolimod, 100, 500, or 1,000 μg/kg Melatonin, 50 or 150 mg/kg NR. **(B)** Protocol 2: For examining NR (50 mg/kg, dissolved in saline) and Melatonin (500 μg/kg, dissolved in 0.99% ethanol) effects on *in vivo* IR injury, rats were randomly assigned to 5 groups: saline, saline + ethanol (0.99%), melatonin, NR, and NR + melatonin. **(C)** Protocol 3: For examining NR effects on *in vivo* IR injury in presence of cangrelor. Rats were randomly treated with saline or NR (50 mg/kg). Cangrelor (60 μg/kg) was infused at 10 min before reperfusion and maintained (360 μg/kg/h) until the end of reperfusion; **(D)** Protocol 4: For examining NR (50 mg/kg) effects on *in vivo* IR injury in presence of propofol. Rats were randomly treated with saline or NR (50 mg/kg). Anesthesia was induced by pentobarbital (1.6 ml/kg), maintenance anesthesia consisted of propofol (12 mg/kg/h), and remifentanil (30 μg/kg/h) instead of FMA.

Myocardial IRI was induced in all rats (Figure 1). Following instrumentation and surgery, all rats were left for 15 min stabilization before start of the experimental protocol. Study medication was infused through the jugular vein at a volume of 1 ml/kg over 3 min (from T0 to T3) and 30 min stabilization followed. Then, the heart was subjected to 25 min of ischemia (LAD coronary artery ligation) followed by 120 min of reperfusion (ligation release). Successful myocardial infarction was confirmed by observation of pallor of the heart and apparent ST segment elevation on the ECG. Because several anesthetic regimens can result in hyperglycemia (24, 25), and high glucose levels can contribute to cardiac IRI, we also monitored blood glucose levels (RAPIDPoint 500, Siemens) for the FMA anesthetic regimen, by sampling blood (0.15 ml) from the carotid artery before start of surgery, 5 min before start of ischemia and 30 min after start of reperfusion.

Following an initial dose response determination of several potential cardio-protective agents, nicotinamide riboside (NR), and melatonin were selected for subsequent testing in combination to achieve maximal reduction of cardiac IRI. Finally, the most effective strategy was examined in the presence of cangrelor or propofol anesthesia. More specifically (Figure 1):

*Protocol 1*: Pilot dose-response curves of empagliflozin (Empa), fingolimod (Fingo), melatonin (Mela), and NR were examined. Initial dosages of the agents were determined from previous work. In short, animal studies have shown SGLT2 inhibitor protection against chronic cardiac failure or acute cardiac I/R injury for dosages between 10 and 30 mg/kg (13). We therefore chose to examine cardioprotection for 10, 25, and 125 mg/kg empagliflozin. For fingolimod we used as starting dosage 1 mg/kg, because that dosage was recently shown to cause infarct size reductions in a large animal model (12). Previous work demonstrated that 10–100 μM melatonin was able to afford acute protection against cardiac IR injury (14, 15). Assuming blood volume of rats to be approximately 7%, our dosages of 100, 500, and 1,000 μg/kg melatonin translate into blood concentrations of ~6, 31, and 62 μM. Yamamoto et al. (17) used 500 mg/kg NMN as NAD precursor to obtain cardioprotection in an *in vivo* mouse IR model. Knowing that NR penetrates cells faster than NMN we decided to start with 50 mg/kg NR.
*Protocol 2*: For examining NR (50 mg/kg, dissolved in saline) and Melatonin (500 μg/kg, dissolved in saline +1% ethanol) effects, rats were randomly assigned to five groups: saline, saline +1% ethanol, melatonin, NR, and NR + melatonin. NR (Niagen) was a gift from Chromadex, Irvine, USA.
*Protocol 3*: For examining NR effects in the presence of cangrelor, rats were randomly assigned to two groups: Saline and NR (50 mg/kg) group. Cangrelor (60 μg/kg) was administrated through the jugular vein starting at 10 min before reperfusion and maintained (360 μg/kg/h) until the end of reperfusion.
*Protocol 4*: For examining NR effects in the presence of propofol, rats were randomly assigned to two groups: Saline and NR (50 mg/kg) group. Instead of FMA, induction of anesthesia was achieved with pentobarbital (80 mg/kg), followed by maintenance anesthesia consisting of propofol (12 mg/kg/h) and remifentanil (30 μg/kg/h) administered through the jugular vein.

### Infarct Size Measurement

After 115 min of reperfusion, 0.8 ml of 25 U.I/ml heparin (Ratiopharm) was infused through the carotid artery cannula. After 120 min of reperfusion, the heart was excised and mounted on a modified Langendorff-apparatus and flushed with heparin (10 U.I/ml) in saline for 2 min. The LAD ligature was then permanently tightened and 0.2% Evans Blue (Sigma) in normal saline was perfused through the heart for 80 s to visualize the area of risk. Intravascular Evans blue was then washed out by perfusion with heparin (10 U.I/ml) in saline for 2 min and hearts were subsequently frozen at −20°C. Frozen hearts were sliced into 2 mm thick transverse sections and incubated in Tris buffered 0.75% 2,3,5-triphenyltetrazolium chloride (TTC, Sigma) solution for 20 min at 37°C and fixed in 4% formalin (Merck) for 24 h at room temperature. Images of each slide were obtained and anonymously forwarded to an external person outside the laboratory for analysis. The infarct size (IS), area at risk (AAR) and left ventricular (LV) area were determined using Image J software by the external person that was blinded to the specific treatments of each heart.

### Lactate Dehydrogenase (LDH) Activity

At 30 min reperfusion, 1 ml blood was obtained from the carotid artery, immediately centrifuged (3 min at 13.400 rpm) and the supernatant (plasma) stored at −80°C for further analysis. LDH activity was determined spectrophotometrically at 25°C, with pyruvate and nicotinamide adenine dinucleotide hydrogen (NADH). Eight hundred microliter LDH assay (containing 0.17 mM NADH) and 12.5 μl pyruvate solution (50 mM) were added to 100 μl plasma sample. The formation of NAD^+^ from NADH was determined over 3 min to obtain LDH activity.

### Exclusion Criteria and Number of Animals Dying During the Experiments

All animals were included in the analysis, except for animals where (1) the AAR was below 10% of the left ventricle, or (2) severe hyperglycemia (>10 mM) occurred at any time during the experiment.

### Statistical Analysis

Data are given as Mean ± SD. The initial sample size *n* = 7 was determined to be able to detect a clinically relevant decrease of 25% in infarct size with an α = 0.05, *SD* = 13.2, and a power of 80%. Shapiro–Wilki was used to test the normality distribution of data. Student's *t*-test or Mann–Whitney test was used to compare the difference between two groups. One-way ANOVA or Kruskal-Wallis test with Bonferroni *post-hoc* test was used for comparing more than two groups. Kruskal-Wallis test with Dunnett *post-hoc* test (saline group as control category) was used for pilot data (protocol 1). Two-way ANOVA for repeated measures with Bonferroni *post-hoc* test was used to compare hemodynamic and blood gas parameters. Analysis was performed using IBM SPSS statistics version 26 (International Business Machines Corp., Armond, NY, USA). Figures were made in GraphPad Prism 8.0 (GraphPad Software, Inc., La Jolla, CA, USA). *P* values are two-sided and considered statistically significant if *P* < 0.05. All tests performed are shown in the Supplementary Statistics file.

## Results

### Excluded and Dead Animals During Experiments

A total of 6 animals were excluded because their AAR <10% of the left ventricle, whereas 1 animal was excluded because of glucose > 10 mM. During the experiments, 11 animals died during the initial anesthesia injection, 13 animals died during the ischemic period, and 13 animals died during the reperfusion period.

### NR and Melatonin Showed Potential for Cardioprotection

**Figure 2:**
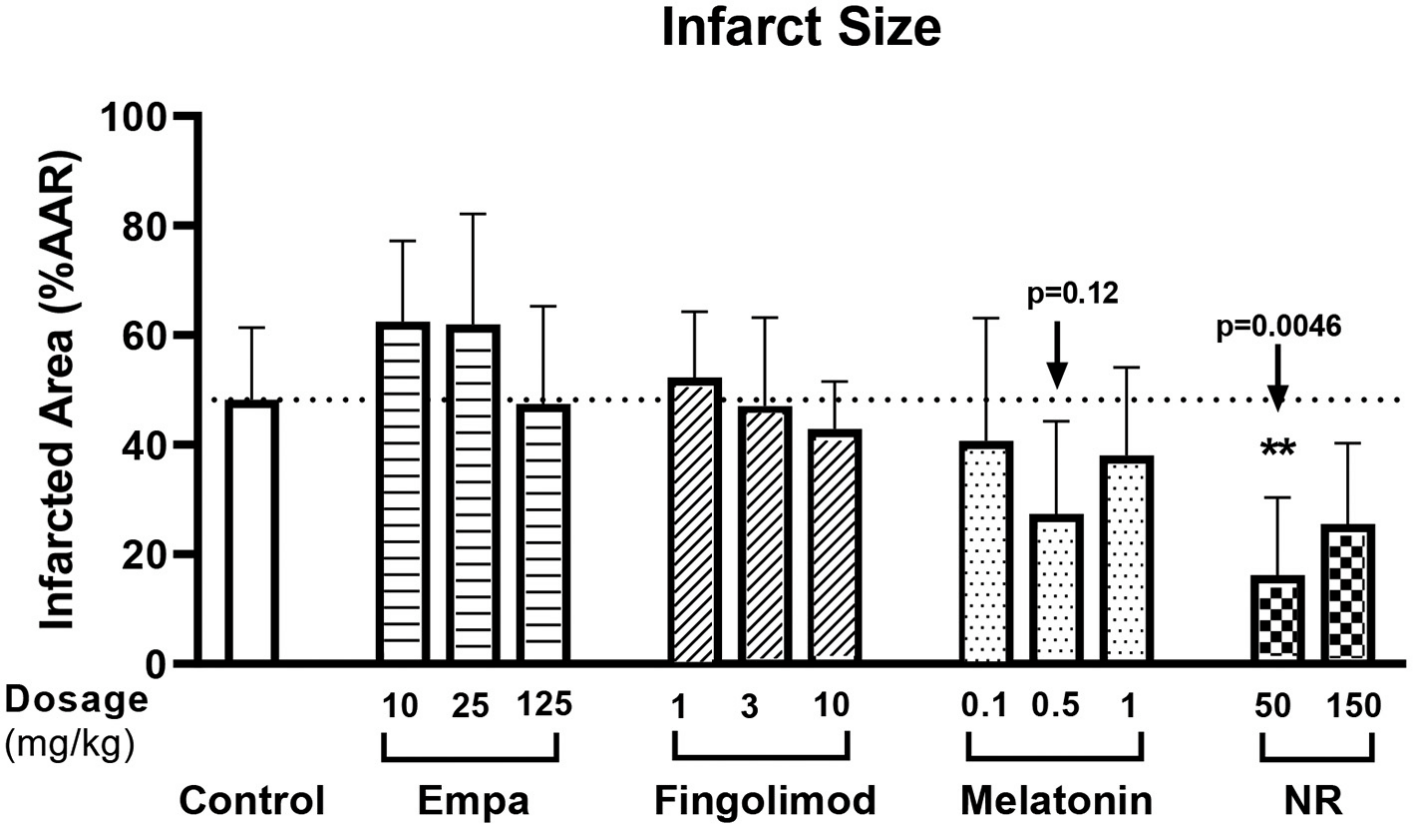
Pilot dose-response of potential cardioprotective agents. The experiment followed protocol 1, rats were randomly subjected to empagliflozin [Empa;10 (*n* = 4), 25 (*n* = 4), 125 (*n* = 5) mg/kg], fingolimod [1 (*n* = 4), 3 (*n* = 4), 10 (*n* = 4) mg/kg], melatonin [100 (*n* = 4), 500 (*n* = 6), 1,000 (*n* = 4) μg/kg], or nicotinamide riboside [NR; 50 (*n* = 5), 150 (*n* = 5) mg/kg] or saline only (control; *n* = 8). Infarct size (IS, %) was related to area at risk (AAR). Data was analyzed by Kruskal-Wallis test with Dunnett *post-hoc* test (saline group as control category).

Pilot dose-response groups were examined for empagliflozin (Empa;10, 25, 125 mg/kg), fingolimod (1, 3, 10 mg/kg), melatonin (100, 500, 1,000 μg/kg), and nicotinamide riboside (NR; 50 and 150 mg/kg) and compared to non-drug treated control animals, using a total of 57 successful experiments (Figure 1A). The pilots indicated that 50 mg/kg NR (*p* = 0.005) or 500 μg/kg melatonin (*p* = 0.12) have potential to reduce infarct size (%) in fentanyl-midazolam anesthetized animals (Control 48 ± 13%, NR 16 ± 14%, melatonin 27 ± 17%, Figure 2). Therefore, 50 mg/kg NR and 500 μg/kg melatonin were selected for further experiments.

### NR Showed Maximal Protection Against *in vivo* Cardiac IR Injury

**Figure 3:**
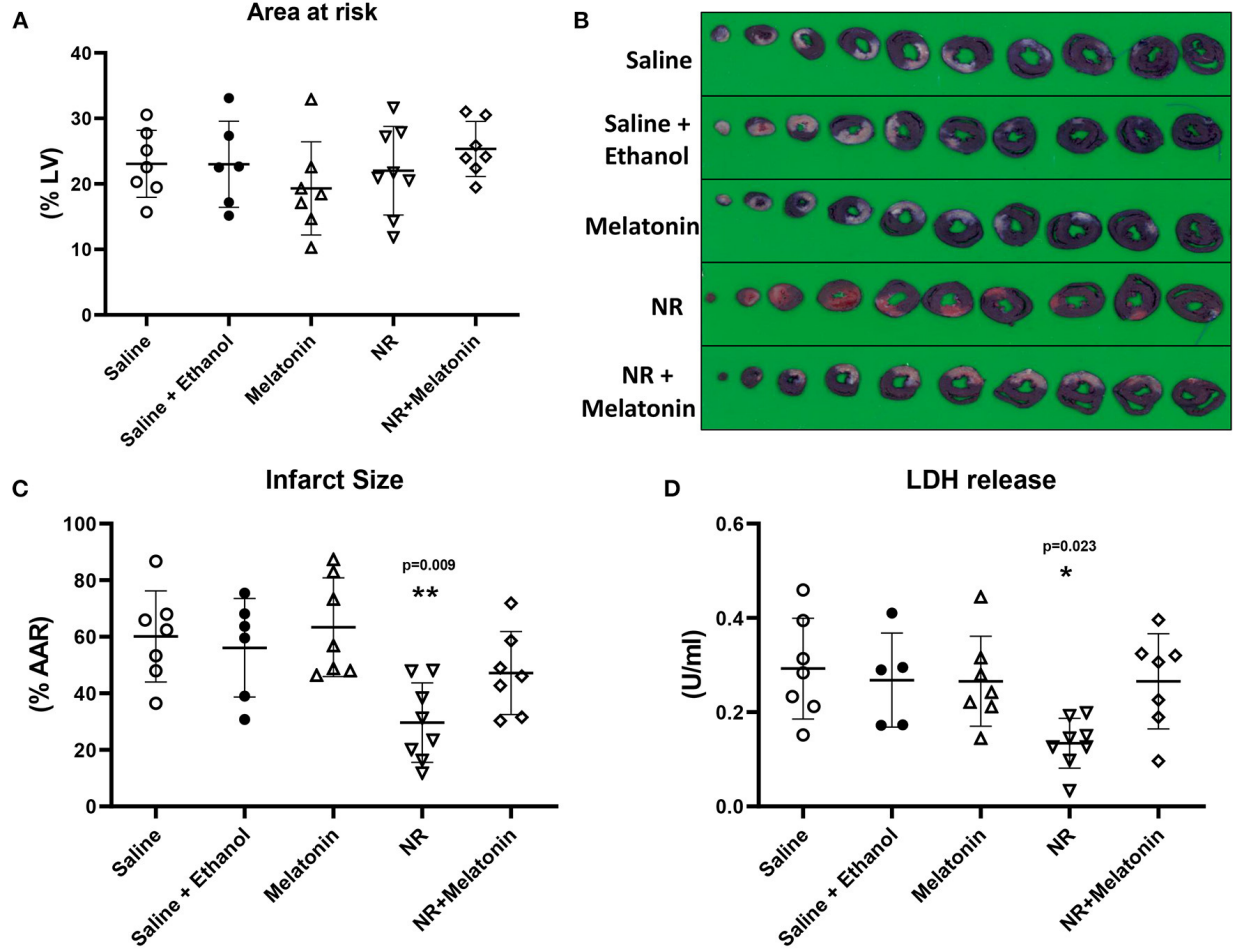
NR treatment alone showed maximal protection against *in vivo* cardiac IR injury. NR (50 mg/kg) treatment (*n* = 8) alone before ischemic insult reduced infarction, but not melatonin (500 μg/kg; *n* = 7) or NR + melatonin (*n* = 7). **(A)** Area at risk (AAR) related to left ventricle area (LV); **(B)** Image of TTC staining; **(C)** Infarct size related to AAR. **(D)** LDH release. *P < 0.05, **P < 0.01 vs. Saline group (*n* = 7), data was analyzed by one-way ANOVA with Bonferroni *post hoc* test.

Baseline physiological variables were similar among groups (Table 1). We firstly determined which treatment provided most profound cardioprotection in acute *in vivo* cardiac IR injury, with NR (50 mg/kg) and melatonin (500 μg/kg) administrated separately or in combination (Figure 1B). The AAR (% of left ventricle) obtained from 25 min LAD occlusion was not significantly different across all five groups, with a clinically relevant mean AAR of 23 ± 6% (*P* = 0.475, Figure 3A). After 25 min I/120 min R, the infarct size (IS) amounted to 60 ± 16% and LDH release amounted to 0.293 ± 0.107 U/ml in the saline group and was unaffected by ethanol (solvent of melatonin) treatment (IS: 56 ± 17%, *P* = 1.000; LDH: 0.268 ± 0.100 U/ml, *P* = 1.000, Figures 3B–D). Only intervention with NR significantly reduced IS by 50% (30 ± 14% vs. Saline, *P* = 0.009, Figures 3B,C) as well as reduced LDH release by 46% (0.134 ± 0.053 U/ml, *P* = 0.023, Figure 3D). No cardioprotection was observed with melatonin (IS: 63 ± 18%, *P* = 1.000; LDH: 0.266 ± 0.096 U/ml, *P* = 1.000, Figures 3B–D), or the combination of melatonin with NR (IS: 47 ± 15%, *P* = 1.000; LDH: 0.266 ± 0.101 U/ml, *P* = 1.000, Figures 3B–D). Contrary to our initial hypothesis, melatonin combined with NR did not show greater protection but even nullified NR's protection.

**Table 1:** Baseline characteristics of the rats in all series.

	**BW (g)**	**MAP (mmHg)**	**HR (bpm)**	***n***
**Series 1**
Saline	339 ± 12	84 ± 13	455 ± 34	7
Saline + Ethanol	354 ± 13	90 ± 11	436 ± 30	6
Melatonin	335 ± 8	78 ± 9	454 ± 29	7
NR	340 ± 23	86 ± 7	463 ± 23	8
NR + Melatonin	343 ± 24	79 ± 9	459 ± 26	7
**Series 2**
Saline + Cangrelor	348 ± 26	67 ± 19	454± 36	7
NR + Cangrelor	332 ± 25	70 ± 16	448 ± 40	8
**Series 3**
Saline + Propofol	354 ± 29	91 ± 38	397 ± 46	6
NR + Propofol	337 ± 19	80 ± 39	359 ± 32	6

*No differences were observed between groups within each series across all the baseline parameters. BW, body weight; MAP, mean arterial pressure; HR, heart rate. Data are presented as mean ± SD, normally distributed data was analyzed by independent t-test or one-way ANOVA followed with Bonferroni multiple comparison. Non-normally distributed data was analyzed by Mann-Whitney U test or Kruskal-Wallis test with Bonferroni multiple comparison*.

### Hemodynamic Variables and Blood Glucose Levels

Mean arterial pressure (MAP) and heart rate (HR) data at five different time points going from baseline to end reperfusion, are presented in Table 2. There was no significant difference in hemodynamic variables between the five experimental groups throughout the experiment. Furthermore, there was no significant change in HR over time for all groups. However, there was a significant decrease in MAP at onset of ischemia (T33 vs. T0, *P* = 0.003), which fully recovered after onset of reperfusion (T58 vs. T33, *P* = 0.0005), these time effects were observed within all five groups.

**Table 2:** Hemodynamic variables throughout the ischemia reperfusion protocol.

	**Baseline**	**Onset of ischemia**	**Onset of reperfusion**	**30 min Reperfusion**	**110 min Reperfusion**
	**T0**	**T33**	**T58**	**T88**	**T168**
	**Mean arterial pressure (mmHg)** ^**^ ***P*** **=** **0.003** ^###^ ***P*** **=** **0.00049**				
Saline	65 ± 14	58 ± 16	68 ± 11	57 ± 12	61 ± 19
Saline + Ethanol	56 ± 16	53 ± 12	61 ± 10	61 ± 11	69 ± 12
Melatonin	69 ± 10	59 ± 10	68 ± 11	64 ± 7	64 ± 11
NR	64 ± 10	51 ± 7	62 ± 10	60 ± 17	61 ± 19
NR + Melatonin	67 ± 10	57 ± 21	67 ± 20	72 ± 26	72 ± 30
	**Heart rate (bpm)**				
Saline	454 ± 48	460 ± 59	474 ± 35	478 ± 27	462 ± 61
Saline + Ethanol	420 ± 48	426 ± 41	417 ± 62	442 ± 66	453 ± 44
Melatonin	439 ± 23	445 ± 39	454 ± 56	469 ± 30	457 ± 25
NR	449 ± 34	430 ± 57	469 ± 31	463 ± 41	468 ± 22
NR+ Melatonin	456 ± 39	430 ± 62	438 ± 38	449 ± 17	458 ± 28

*T, time in minutes. Data are presented as mean ± SD, analyzed by two-way Repeated Measures ANOVA applying to the Bonferroni multiple comparisons test when relevant*.

** *p < 0.01 (T33 vs. T0 within group)*.

### *p < 0.001 (T58 vs. T33 within group)*.

Although NR and NR + melatonin increased mean arterial pressure during drug infusion (Additional file 1: Supplementary Figure 1), blood pressure was quickly normalized 2 min after infusion of NR.

**Figure 4:**
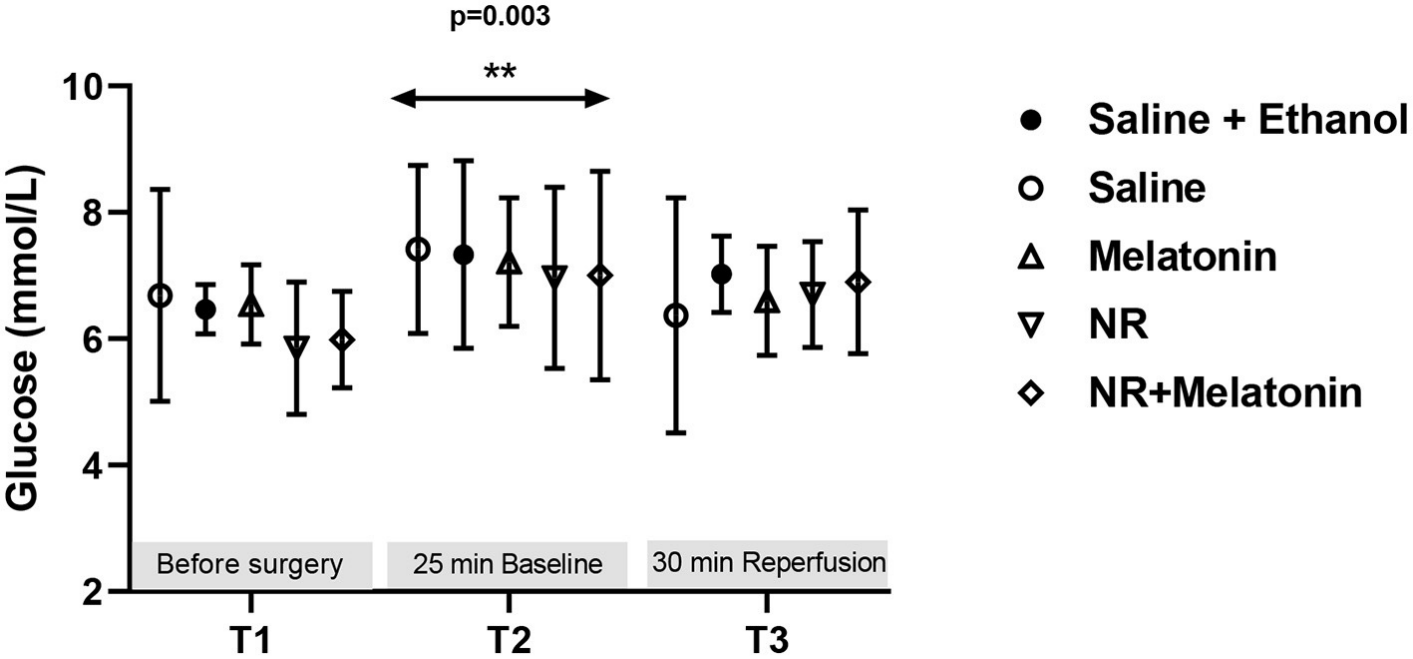
Blood glucose level from the 5 experimental groups at three time points. Glucose (mmol/L) levels were detected before surgery, at 25 min baseline and 30 min reperfusion. **P < 0.01 vs. T1, data was analyzed using two-way Repeated Measures ANOVA applying the Bonferroni multiple comparisons test when relevant. All groups *n* = 6–8 experiments.

Glucose levels were detected throughout the experiment, indicating no treatment effects (Figure 4). There was an increased glucose level (*P* = 0.003, T2 vs. T1, Figure 4) immediately following cardiac surgery which decreased to baseline levels at 30 min reperfusion. Most importantly, almost all glucose levels were below 10 mM, demonstrating that hyperglycemia does not develop with fentanyl-midazolam-acepromazine anesthesia in this preclinical model.

### NR Treatment Protects Against *in vivo* Cardiac IR Injury in Presence of Cangrelor

**Figure 5:**
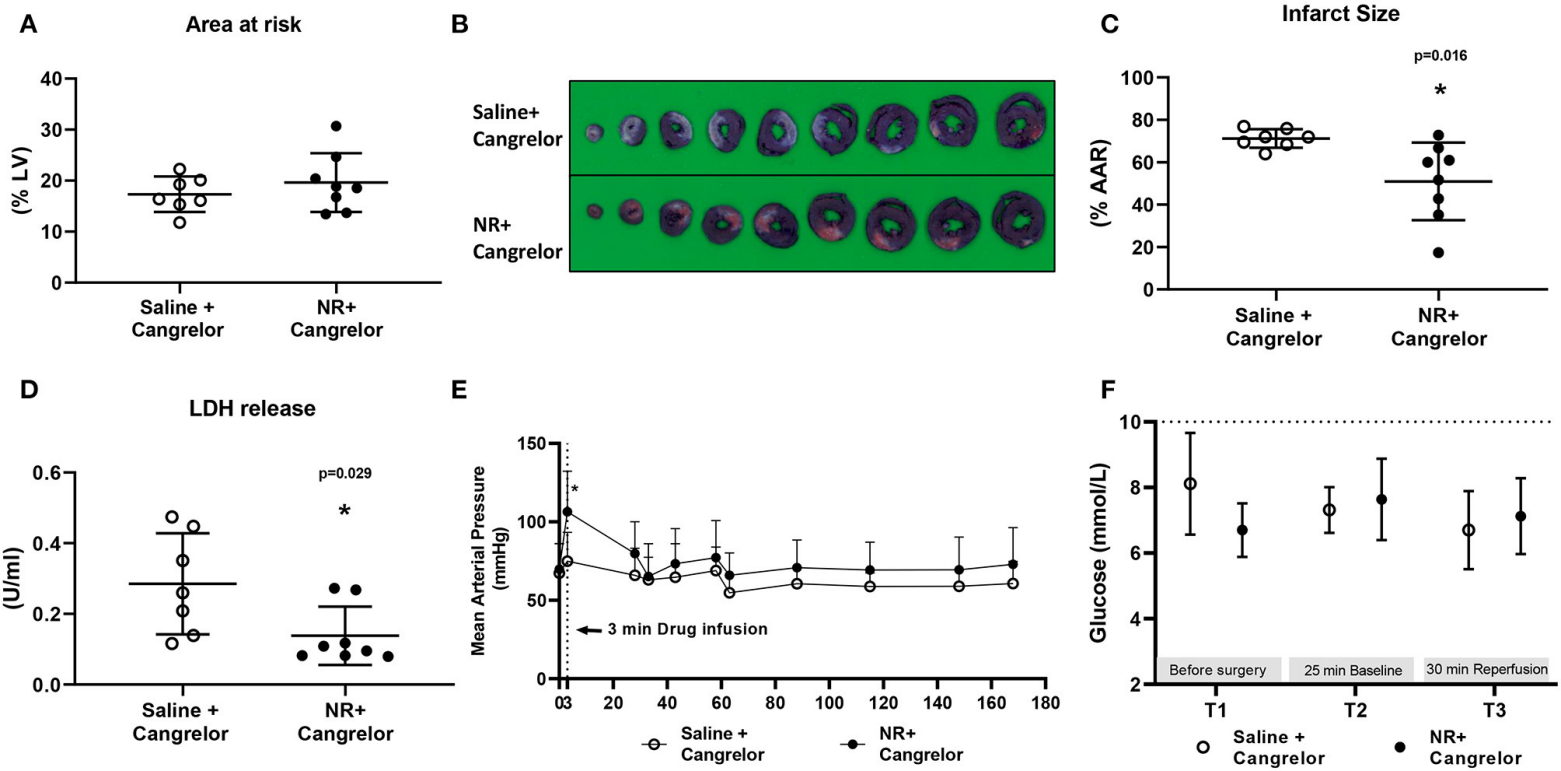
NR effects against *in vivo* cardiac IR injury in presence of cangrelor. NR treatment reduced infarction in the presence of cangrelor, a common clinically used P2Y12 inhibitor. **(A)** Area at risk (AAR) related to left ventricle area (LV); **(B)** Image of TTC staining; **(C)** Infarct size related to AAR; **(D)** LDH release; **(E)** Mean arterial pressure (MAP, mmHg) throughout the whole protocol; **(F)** glucose level at three time point. *P < 0.05 vs. Saline group, AAR/LV and IS/AAR data was analyzed by Student's *t*-test; LDH data was analyzed by Mann-Whitney *U* test; MAP and glucose level were analyzed using two-way Repeated Measures ANOVA applying the Bonferroni multiple comparisons test when relevant. Saline group (*n* = 7); NR group (*n* = 8).

To determine whether NR treatment can maintain its benefit in several clinically relevant conditions in our *in vivo* cardiac IR model, we first examined NR protection in presence of cangrelor, a fast-acting reversible P2Y_12_ inhibitor (Figure 1C). Baseline physiological variables were summarized and were similar for both groups (Table 1). No differences in AAR (%LV) were observed between the two groups (Figure 5A). In the presence of cangrelor, NR still significantly reduced infarct size (Saline 71 ± 4%, NR 51 ± 18%, *P* = 0.016, Figures 5B,C) and LDH release (Saline 0.285 ± 0.143 U/ml, NR 0.138 ± 0.083 U/ml, *P* = 0.029, Figure 5D). A similar transient elevation of blood pressure was observed during 3 min of NR infusion (Saline 75 ± 18 mmHg, NR 106 ± 26 mmHg, *P* = 0.019, Figure 5E) as in the absence of cangrelor. MAP was similar between groups throughout the remaining part of the experiment. Hyperglycemia was not observed in the presence of FMA plus cangrelor (Figure 5F).

### NR Treatment Lost Cardiac Protection in Presence of Propofol

**Figure 6:**
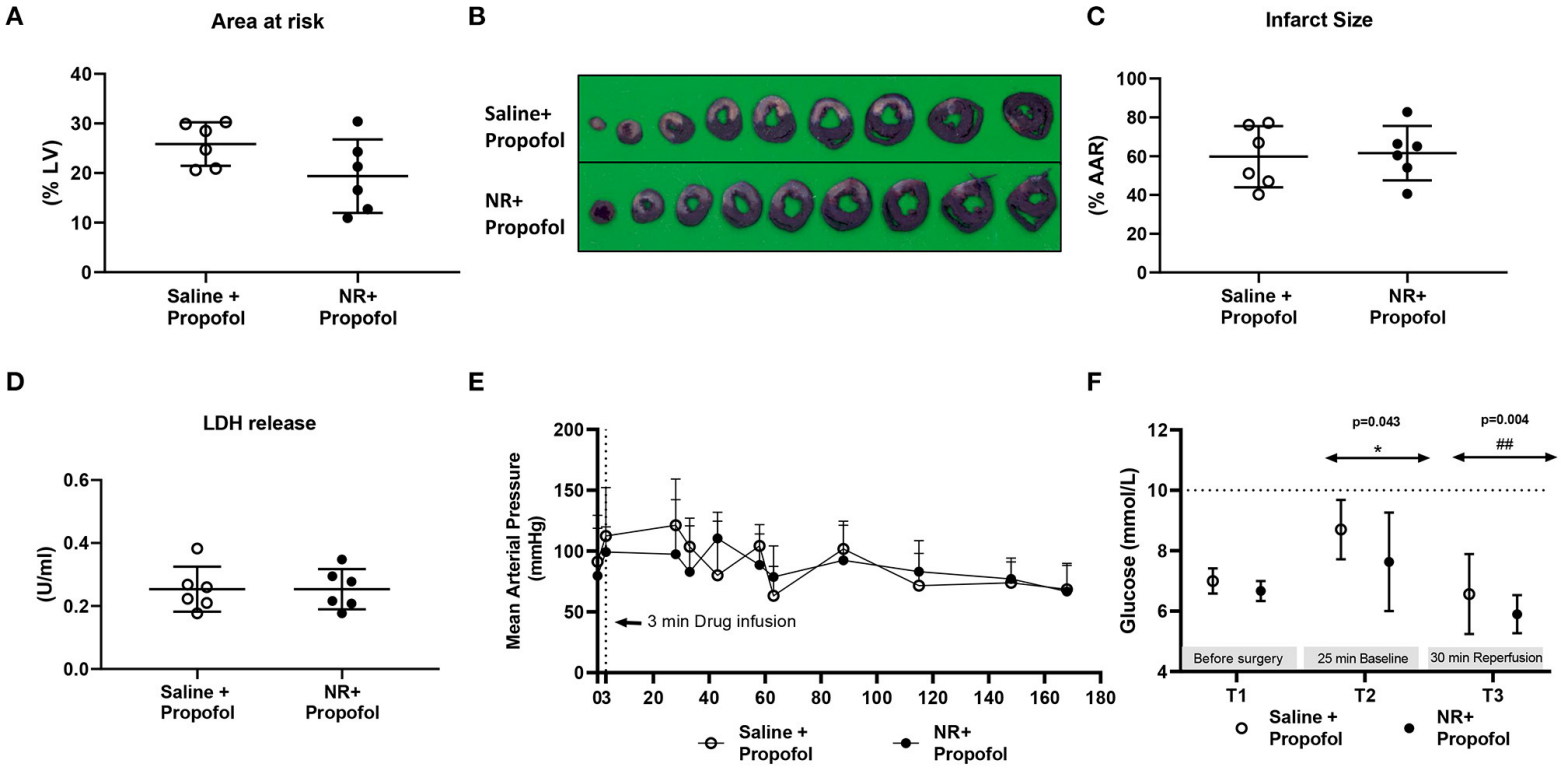
NR lost protection against *in vivo* cardiac IR injury in presence of propofol. NR treatment failed to reduce infarction in the presence of propofol, a common clinically used anesthesia. **(A)** Area at risk (AAR) related to left ventricle area (LV); **(B)** Image of TTC staining; **(C)** Infarct size related to AAR; **(D)** LDH release; **(E)** Mean arterial pressure (mmHg) throughout the whole protocol; **(F)** glucose level at three time point. Data was analyzed by Student's *t*-test; *P < 0.05 vs. T1, ^##^*P* < 0.01 vs. T2, MAP and glucose level were analyzed using two-way Repeated Measures ANOVA applying the Bonferroni multiple comparisons test when relevant. Saline group (*n* = 6); NR group (*n* = 6).

Furthermore, we examined NR protection in the presence of propofol, a frequently used hypnotic in cardiac surgery (Figure 1D). Baseline physiological variables were summarized in Table 1. There were no significant differences between groups (Table 1). The AAR (%LV) was similar between two groups (Figure 6A). However, the presence of propofol completely abrogated NR protective effects against IR induced cell death [IS: Saline 60 ± 16%, NR 62 ± 14%, *P* = 0.839; LDH release (Saline 0.253 ± 0.072 U/ml, NR 0.254 ± 0.064 U/ml, *P* = 0.999, Figures 6B–D). Interestingly, propofol anesthesia also abrogated the temporarily blood pressure elevation observed with NR infusion in the other series (saline 113 ± 40 mmHg, NR 99 ± 21 mmHg *P* = 0.489); no differences in MAP between the two groups throughout the experiment were observed (Figure 6E). Glucose levels were detected throughout the experiment, indicating no treatment effects (Figure 6F). There was an increased glucose level (*P* = 0.043, T2 vs. T1, Figure 6F) immediately following cardiac surgery which decreased to baseline levels at 30 min reperfusion level (*P* = 0.301, T3 vs. T1; *P* = 0.004, T3 vs. T2 Figure 6F). Hyperglycemia was not observed with fentanyl-propofol anesthesia throughout the experiment (Figure 6F).

## Discussion

To improve translatability and start building a multitarget protective strategy against acute cardiac IRI, we have examined the efficacy of various previously reported cardioprotective compounds in the context of a clinically frequently used anesthetic with further testing in the presence of a platelet antagonist or propofol anesthesia. The major results concerning acute pre-ischemic administration of the compounds suggest that (1) the NAD^+^ precursor NR is most suitable to become part of a multitarget cardioprotective strategy because it reduces cardiac IRI in the presence of fentanyl, benzodiazepines and a P2Y_12_ inhibitor, (2) NR should not be used as cardioprotective agent in the presence of propofol, and (3) empagliflozin, fingolimod and melatonin are not recommended as cardioprotective agents, at least in the presence of fentanyl and benzodiazepines. Employing fentanyl with midazolam or propofol for rat surgical anesthesia is not associated with hyperglycemia, in contrast to other often recommended anesthetic regimens (e.g., ketamine-xylazine) for preclinical models of cardioprotection (27).

### NR and Cardiac IRI

Previous work demonstrated reduced cardiac infarct size for both *in vivo* and *in vitro* conditions employing acute administration of the NAD^+^ precursor NMN, or NAD^+^ itself (17–19). However, in these *in vivo* studies, pentobarbital was used as main hypnotic. Pentobarbital is not commonly used in humans during PCI procedures, questioning translatability of these results. The current study shows that NR treatment also reduces infarct size *in vivo*. We specifically choose NR because this compound has been suggested as the optimal precursor for boosting muscle NAD^+^ content (28, 29). In addition, protection was also observed in the presence of clinically used anesthetic agents what enables its use in the clinical setting. Intravenous infusion of NR caused a significant increase in blood pressure that was dissipated 2 min after termination of the infusion. A sharp large increase in blood pressure, even short-lived, may be unwanted in the clinical scenario, and should therefore be avoided. A slower infusion and/or more dilution of the compound may possibly mitigate this large increase. NR effects on blood pressure with i.v. administration have not yet been reported and underlying mechanisms are unknown. Possibly a large dosage administered in a short time may activate GPR109a-induced calcium release resulting in temporary blood pressure increases (30), although NR dosages <1 mM are without effect on GPR109a (28). Further studies are warranted to explore the mechanisms of NR-induced acute blood pressure elevations.

#### NR in the Presence of Melatonin

Surprisingly, melatonin abrogated NR protection in our model. We were unable to find other research that has examined this interaction between a NAD precursor and melatonin within one study. The most likely explanation may be found in literature that has reported that melatonin switch metabolism from aerobic glycolysis toward mitochondrial TCA cyclus activity (31), indicating that melatonin can inhibit glycolysis. One of the suggested protective mechanisms of NAD suppletion in the setting of acute cardiac I/R injury is the activation of glycolysis (19). In addition, glycolysis is often needed for cardioprotective interventions (4). Additionally, melatonin was also reported as inhibitor of autophagy (32), whereas protection by NAD precursor administration was suggested to be mediated through activation of autophagy (18). Therefore, it is possible that melatonin abrogated NR protective actions through glycolysis and/or autophagy inhibition, however, so far, this is purely hypothetical. Further research will be necessary to explore this possible interaction between melatonin and NAD administration.

### NR in the Presence of Cangrelor or Propofol

The use of direct-acting, quick onset, P2Y_12_ antagonists has demonstrated cardiovascular benefits and has become standard treatment during and after PCI procedures (33, 34). Preclinical studies showed that these antagonists confer acute cardioprotection through cardiac activation of pro-survival kinases of the reperfusion injury salvage kinase (RISK) pathway (20, 21). Opioids also protect through the RISK pathway and the JAK/STAT pathway (8, 35, 36). The standard clinical use of P2Y_12_ antagonists and opioids therefore suggest that cardioprotective interventions for which underlying mechanisms entail the activation of RISK and JAK/STAT pathway may be less likely to confer successful clinical translation toward PCI procedures when these pathways are already largely activated by the background clinical therapeutic agents. Although the underlying infarct-reducing mechanisms of NAD^+^ precursors have not been fully unraveled, activation of sirtuin 1, mitophagy and glycolysis has been suggested (17–19). NR and NMN activate sirtuin 1 and glycolysis by increasing the NAD^+^/NADPH redox couple (19). Stimulating sirtuin 1 (37) and glycolysis (4) offer protection against IRI. In a chronic model of I/R injury (days instead of hours), it was recently shown that NAD^+^ treatment protects through restoration of transcription factor EB-mediated lysosomal autophagic flux (18). Protection through increased expression of transcription factors seems unlikely for the acute protection by NR observed in the present study, thus it remains to be examined whether activated autophagy contributed to NR-mediated acute protection. Most importantly, the NR-induced stimulation of sirtuin 1 and glycolysis seems to be independent of the RISK and Jak/STAT3 pathways, explaining its potential for clinical translation.

NR was ineffective in the presence of propofol, adding to propofol's known abrogation of cardioprotective signaling. Propofol was already reported to abolish high dose-folic acid cardioprotection, remote ischemic preconditioning or intralipid-postconditioning. Loss of cardioprotection was explained by the radical scavenging properties of propofol (22, 23, 38). It is likely that the attenuation of reactive oxygen species (ROS) by propofol also contributes to the loss of NR protection, knowing that increased ROS during reperfusion is a common end-effector of cardiac I/R injury (2, 3). It is therefore anticipated that the presence of propofol will make it difficult for any protective intervention to remain effective. It should thereby be noted that although propofol in itself bares protective properties against cardiac I/R injury in isolated hearts when compared to no anesthetics (39, 40), this protective effect of propofol is often nullified when compared to other anesthetics (22, 23, 38).

### SGLT2 Inhibitors, Melatonin, and Fingolimod and Cardiac IRI

The present study suggests that acute treatments with SGLT2i's, melatonin or fingolimod are ineffective in reducing infarct size in the presence of a fentanyl-midazolam anesthetic regimen. Although, this is in support of most studies that have employed SGLT2i's (13), it is somewhat more surprising for melatonin and fingolimod, for which most preclinical studies did report protective effects. However, it is in line with the first clinical study of melatonin treatment in PCI patients showing no beneficial (even detrimental) effects of melatonin (41, 42). We are also unaware of preclinical studies that had examined melatonin or fingolimod cardioprotective efficacy in animals anesthetized with fentanyl-midazolam, at least offering one possible explanation as to the clinical failing of melatonin. Melatonin's protective effect was reported to be, at least partly, mediated through activation of the RISK and JAK/STAT3 pathway (43), which pathway may have already been activated by the presence of fentanyl.

## Limitations

The main goal of the present study was to explore whether previously reported cardio-protective agents maintain protection on a background of clinical relevant therapeutic agents. Therefore, and as a limitation, no explorations of the underlying mechanism of NR protection, or why NR lost protection in the presence of propofol, were performed. Further studies will be necessary to elucidate these mechanisms. It should be noted that other clinical agents and patient characteristics, that were not tested in the current study, also may affect the heart's resistance against IRI. Among them are medications such as heparin, statins, β blockers, angiotensin-converting enzyme inhibitors and comorbidities such as hyperglycemia, diabetes, obesity, hypertension, and aging. These co-medications and comorbidities will also have to be tested with NR before moving to the clinical setting. In addition, we did not examine NR in combination with other cardioprotective strategies that were recently shown to maintain their protective efficacy in the presence of clinically used drugs, such as caspase inhibitors (44), hypothermia (21) and/or an inhibitor of the sodium/hydrogen exchanger (21). The challenge will be to rationalize the best choice of these different agents, possibly by ensuring that each agent activates a separate survival pathway, to develop a multitarget therapy for successful clinical translation. Finally, in the present study we first wanted to examine cardioprotective effects of various drugs in the context of a clinically used background therapy when these compounds were provided before start of ischemia, mimicking conditions present during elective cardiac surgery. Further research is necessary to test efficacy of NR, together with other compounds, when administered just before start of reperfusion, enabling use for PCI.

## Conclusions

To improve effective translation from preclinical models to the clinical setting, there is a need to resemble common clinical modalities present during clinical interventions (e.g., cardiac surgery or PCI) in our animal models. In the present study we showed that in the presence of a frequently clinically used anesthesia regimen and anticoagulation therapy, acute administration of nicotinamide riboside (NR), but not empagliflozin, melatonin or fingolimod, reduced acute cardiac IRI, suggesting that NR is suitable for a multitarget cardioprotective strategy for clinical treatment during PCI procedures.

## Data Availability Statement

The original contributions presented in the study are included in the article/Supplementary Material, further inquiries can be directed to the corresponding authors.

## Ethics Statement

The animal study was reviewed and approved by Animal Ethics Committee of the Academic Medical Centre, Amsterdam, The Netherlands.

## Author Contributions

YX and CZ designed the research and drafted the manuscript. YX, DB, PP, QW, and RN performed the experiments and the data analysis. RN and MH revised the manuscript. All authors reviewed the results and approved the final version of the manuscript.

## Conflict of Interest

The authors declare that the research was conducted in the absence of any commercial or financial relationships that could be construed as a potential conflict of interest.

## Publisher's Note

All claims expressed in this article are solely those of the authors and do not necessarily represent those of their affiliated organizations, or those of the publisher, the editors and the reviewers. Any product that may be evaluated in this article, or claim that may be made by its manufacturer, is not guaranteed or endorsed by the publisher.

## Abbreviations

IRI: Ischemia-reperfusion injury
Fingo: Fingolimod
Empa: Empagliflozin
Mela: Melatonin
NR: Nicotinamide riboside
IS: Infarct size
LAD: Left ascending coronary
PCI: Percutaneous coronary interventions
SGLT2: Sodium glucose cotransporter 2
NMN: Nicotinamide mononucleotide
TIVA: Total intravenous anesthesia
ECG: Electrocardiograms
TTC, 2, 3: 5-triphenyltetrazolium chloride
AAR: Area at risk
LV: Left ventricle
MAP: Mean arterial pressure
HR: Heart rate
RISK: Reperfusion injury salvage kinase
ROS: Reactive oxygen species.
