# Supplementary material for: Cardioprotecive Properties of Known Agents in Rat Ischemia-Reperfusion Model Under Clinically Relevant Conditions: Only the NAD Precursor Nicotinamide Riboside Reduces Infarct Size in Presence of Fentanyl, Midazolam and Cangrelor, but Not Propofol
Source: Front Cardiovasc Med. 2021 Aug 30;8:712478. doi: 10.3389/fcvm.2021.712478 (PMC8435675; doi:10.3389/fcvm.2021.712478)
Supplement: Supplementary file 1 [file Data_Sheet_1.PDF]

Figure 2,

As reviewers suggested , we performed the non-parametric test (independent-sample kruskal-wallis test) due to the small sample size, each drug dosage compared to control group, the statistic results are shown below:

Empa						
Dunn's mu	Mean rank	Significant	Summary	Adjusted F	A-?	
Control vs.	-5.188	No	ns	0.5161	B	10mg/kg
Control vs.	-4.188	No	ns	0.8108	C	25mg/kg
Control vs.	0.4125	No	ns	>0.9999	D	125mg/kg

Fingolimod						
Dunn's mu	Mean rank	Significant	Summary	Adjusted P Value	A-?	
Control vs.	-1.875	No	ns	>0.9999	E	1mg/kg
Control vs.	0.125	No	ns	>0.9999	F	3mg/kg
Control vs.	2.375	No	ns	>0.9999	G	10mg/kg

Melatonon						
Dunn's mu	Mean rank	Significant	Summary	Adjusted F	A-?	
Control vs.	1.375	No	ns	>0.9999	H	100mg/kg
Control vs.	7.208	No	ns	0.1195	I	500mg/kg
Control vs.	3.625	No	ns	>0.9999	J	1000mg/kg

NR						
Dunn's mu	Mean rank	Significant	Summary	Adjusted F	A-?	
Control vs.	9.275	Yes	**	0.0046	K	50mg/kg
Control vs.	6.475	No	ns	0.0668	L	150mg/kg

Figure 3.

Step 1: Results of normality test:

Tests of Normality

		Kolmogorov-Smirnov[a]			Shapiro-Wilk		
	Group_S2_NR+Mel	Statistic	df	Sig.	Statistic	df	Sig.
LV_S2	Saline	.281	7	.100	.918	7	.454
	Saline + Ethanol	.314	6	.066	.872	6	.233
	Melatonin	.242	7	.200*	.919	7	.463
	NR	.156	8	.200*	.941	8	.617
	NR+Melatonin	.286	7	.086	.780	7	.026
AAR_LV_S2	Saline	.133	7	.200*	.985	7	.979
	Saline + Ethanol	.187	6	.200*	.958	6	.804
	Melatonin	.215	7	.200*	.929	7	.539
	NR	.163	8	.200*	.954	8	.756
	NR+Melatonin	.182	7	.200*	.932	7	.564
IS_AAR_S2	Saline	.170	7	.200*	.982	7	.968
	Saline + Ethanol	.245	6	.200*	.912	6	.452
	Melatonin	.226	7	.200*	.853	7	.130
	NR	.171	8	.200*	.920	8	.428
	NR+Melatonin	.164	7	.200*	.946	7	.696

*. This is a lower bound of the true significance.

a. Lilliefors Significance Correction

Tests of Normality

	group5	Kolmogorov-Smirnov[a] Statistic	df	Sig.	Shapiro-Wilk Statistic	df	Sig.
LDH_5group	Saline	.139	7	.200*	.973	7	.919
	Saline+Ethanol	.230	5	.200*	.889	5	.352
	Melatonin	.168	7	.200*	.938	7	.618
	NR	.194	8	.200*	.936	8	.569
	NR+Melatonin	.229	7	.200*	.949	7	.723

*. This is a lower bound of the true significance.

a. Lilliefors Significance Correction

Step 2: Due to all groups are normality distributed, then test Homogeneity of Variances:

Test of Homogeneity of Variances

		Levene Statistic	df1	df2	Sig.
AAR_LV_S2	Based on Mean	.319	4	30	.863
	Based on Median	.301	4	30	.875
	Based on Median and with adjusted df	.301	4	25.059	.875
	Based on trimmed mean	.310	4	30	.869
IS_AAR_S2	Based on Mean	.381	4	30	.820
	Based on Median	.143	4	30	.965
	Based on Median and with adjusted df	.143	4	26.955	.965
	Based on trimmed mean	.364	4	30	.832

Test of Homogeneity of Variances

		Levene Statistic	df1	df2	Sig.
LDH_5group	Based on Mean	1.006	4	29	.420
	Based on Median	.593	4	29	.670
	Based on Median and with adjusted df	.593	4	24.679	.671
	Based on trimmed mean	.990	4	29	.429

Step 3: all groups are normality distributed, and equal Homogeneity of Variances, so use one-way ANOVA with Bonferroni post-hoc test:

ANOVA

				Sum of Squares	df	Mean Square	F	Sig.
AAR_LV_S2	Between Groups	(Combined)		132.635	4	33.159	.902	.475
		Linear Term	Unweighted	8.932	1	8.932	.243	.626
			Weighted	8.970	1	8.970	.244	.625
			Deviation	123.665	3	41.222	1.122	.356
	Within Groups			1102.676	30	36.756		
	Total			1235.311	34			
IS_AAR_S2	Between Groups	(Combined)		5549.868	4	1387.467	5.483	.002
		Linear Term	Unweighted	1910.459	1	1910.459	7.550	.010
			Weighted	2085.272	1	2085.272	8.240	.007

IS_AAR_S2	Bonferroni	Saline	Saline + Ethanol	4.00557	8.85025	1.000	-22.8089	30.8201
			Melatonin	-3.25587	8.50305	1.000	-29.0184	22.5066
			NR	30.46150*	8.23304	.009	5.5170	55.4060
			NR+Melatonin	12.94740	8.50305	1.000	-12.8151	38.7099
		Saline + Ethanol	Saline	-4.00557	8.85025	1.000	-30.8201	22.8089
			Melatonin	-7.26144	8.85025	1.000	-34.0759	19.5530
			NR	26.45593*	8.59116	.044	.4264	52.4854
			NR+Melatonin	8.94183	8.85025	1.000	-17.8727	35.7563
		Melatonin	Saline	3.25587	8.50305	1.000	-22.5066	29.0184
			Saline + Ethanol	7.26144	8.85025	1.000	-19.5530	34.0759
			NR	33.71737*	8.23304	.003	8.7729	58.6618
			NR+Melatonin	16.20327	8.50305	.663	-9.5592	41.9658
		NR	Saline	-30.46150*	8.23304	.009	-55.4060	-5.5170
			Saline + Ethanol	-26.45593*	8.59116	.044	-52.4854	-.4264
			Melatonin	-33.71737*	8.23304	.003	-58.6618	-8.7729
			NR+Melatonin	-17.51410	8.23304	.417	-42.4586	7.4304
		NR+Melatonin	Saline	-12.94740	8.50305	1.000	-38.7099	12.8151
			Saline + Ethanol	-8.94183	8.85025	1.000	-35.7563	17.8727
			Melatonin	-16.20327	8.50305	.663	-41.9658	9.5592
			NR	17.51410	8.23304	.417	-7.4304	42.4586

Multiple Comparisons

Dependent Variable: LDH_5group

	(I) group5	(J) group5	Mean Difference (I-J)	Std. Error	Sig.	95% Confidence Interval Lower Bound	95% Confidence Interval Upper Bound
Bonferroni	Saline	Saline+Ethanol	.02453	.05370	1.000	-.1386	.1877
		Melatonin	.02688	.04902	1.000	-.1220	.1758
		NR	.15860*	.04746	.023	.0144	.3028
		NR+Melatonin	.02702	.04902	1.000	-.1219	.1759
	Saline+Ethanol	Saline	-.02453	.05370	1.000	-.1877	.1386
		Melatonin	.00236	.05370	1.000	-.1608	.1655
		NR	.13407	.05228	.158	-.0248	.2929
		NR+Melatonin	.00250	.05370	1.000	-.1606	.1656
	Melatonin	Saline	-.02688	.04902	1.000	-.1758	.1220
		Saline+Ethanol	-.00236	.05370	1.000	-.1655	.1608
		NR	.13171	.04746	.096	-.0125	.2759
		NR+Melatonin	.00014	.04902	1.000	-.1488	.1491
	NR	Saline	-.15860*	.04746	.023	-.3028	-.0144
		Saline+Ethanol	-.13407	.05228	.158	-.2929	.0248
		Melatonin	-.13171	.04746	.096	-.2759	.0125
		NR+Melatonin	-.13158	.04746	.096	-.2758	.0126
	NR+Melatonin	Saline	-.02702	.04902	1.000	-.1759	.1219
		Saline+Ethanol	-.00250	.05370	1.000	-.1656	.1606
		Melatonin	-.00014	.04902	1.000	-.1491	.1488
		NR	.13158	.04746	.096	-.0126	.2758

Figure 4

We used two-way Repeated Measures ANOVA applying the Bonferroni multiple comparisons test,

By doing this we first perform the Mauchly's test of Sphericity, then we go further with tests of within-subjects effects and Pairwise comparisons, see the results below:

Mauchly's Test of Sphericity[a]

Measure: MEASURE_1

Within Subjects Effect	Mauchly's W	Approx. Chi-Square	df	Sig.	Epsilon[b] Greenhouse-Geisser	Huynh-Feldt	Lower-bound
time	.955	1.254	2	.534	.957	1.000	.500

Tests the null hypothesis that the error covariance matrix of the orthonormalized transformed dependent variables is proportional to an identity matrix.

a. Design: Intercept + Group_BGA
Within Subjects Design: time

b. May be used to adjust the degrees of freedom for the averaged tests of significance. Corrected tests are displayed in the Tests of Within-Subjects Effects table.

Tests of Within-Subjects Effects

Measure: MEASURE_1

Source		Type IV Sum of Squares	df	Mean Square	F	Sig.
time	Sphericity Assumed	12.467	2	6.233	7.987	.001
	Greenhouse-Geisser	12.467	1.913	6.516	7.987	.001
	Huynh-Feldt	12.467	2.000	6.233	7.987	.001
	Lower-bound	12.467	1.000	12.467	7.987	.009
time * Group_BGA	Sphericity Assumed	5.753	8	.719	.921	.506
	Greenhouse-Geisser	5.753	7.653	.752	.921	.503
	Huynh-Feldt	5.753	8.000	.719	.921	.506
	Lower-bound	5.753	4.000	1.438	.921	.465
Error(time)	Sphericity Assumed	43.704	56	.780		
	Greenhouse-Geisser	43.704	53.569	.816		
	Huynh-Feldt	43.704	56.000	.780		
	Lower-bound	43.704	28.000	1.561		

Pairwise Comparisons

Measure: MEASURE_1

(I) time	(J) time	Mean Difference (I-J)	Std. Error	Sig.[b]	95% Confidence Interval for Difference[b] Lower Bound	Upper Bound
1	2	-.877*	.235	.003	-1.476	-.278
	3	-.438	.226	.187	-1.013	.137
2	1	.877*	.235	.003	.278	1.476
	3	.439	.195	.099	-.059	.937
3	1	.438	.226	.187	-.137	1.013
	2	-.439	.195	.099	-.937	.059

Based on estimated marginal means

*. The mean difference is significant at the .05 level.

b. Adjustment for multiple comparisons: Bonferroni.

Pairwise Comparisons

Measure: MEASURE_1

(I) Group_BGA	(J) Group_BGA	Mean Difference (I-J)	Std. Error	Sig.[a]	95% Confidence Interval for Difference[a]	
					Lower Bound	Upper Bound
Saline	Saline+Ethanol	.050	.548	1.000	-1.620	1.721
	Melatonin	.038	.501	1.000	-1.487	1.563
	NR	.243	.501	1.000	-1.282	1.768
	NR+Melatonin	.195	.501	1.000	-1.330	1.721
Saline+Ethanol	Saline	-.050	.548	1.000	-1.721	1.620
	Melatonin	-.012	.548	1.000	-1.683	1.658
	NR	.192	.548	1.000	-1.478	1.863
	NR+Melatonin	.145	.548	1.000	-1.526	1.816
Melatonin	Saline	-.038	.501	1.000	-1.563	1.487
	Saline+Ethanol	.012	.548	1.000	-1.658	1.683
	NR	.205	.501	1.000	-1.321	1.730
	NR+Melatonin	.157	.501	1.000	-1.368	1.682
NR	Saline	-.243	.501	1.000	-1.768	1.282
	Saline+Ethanol	-.192	.548	1.000	-1.863	1.478
	Melatonin	-.205	.501	1.000	-1.730	1.321
	NR+Melatonin	-.048	.501	1.000	-1.573	1.478
NR+Melatonin	Saline	-.195	.501	1.000	-1.721	1.330
	Saline+Ethanol	-.145	.548	1.000	-1.816	1.526
	Melatonin	-.157	.501	1.000	-1.682	1.368
	NR	.048	.501	1.000	-1.478	1.573

Based on estimated marginal means

a. Adjustment for multiple comparisons: Bonferroni.

Figure 5a-d.

Step 1: Test of normality:

Tests of Normality

	Group_S3_can	Kolmogorov-Smirnov[a]			Shapiro-Wilk		
		Statistic	df	Sig.	Statistic	df	Sig.
LV_S3C	Saline+cangrelor	.204	7	.200*	.928	7	.533
	NR+cangrelor	.290	8	.047	.815	8	.042
AAR_S3C	Saline+cangrelor	.177	7	.200*	.973	7	.917
	NR+cangrelor	.196	8	.200*	.915	8	.388
IS_S3C	Saline+cangrelor	.144	7	.200*	.961	7	.827
	NR+cangrelor	.190	8	.200*	.946	8	.668

Tests of Normality

	group_Cangrelor	Kolmogorov-Smirnov[a] Statistic	df	Sig.	Shapiro-Wilk Statistic	df	Sig.
LDH_Cangrelor	Saline+Cangrelor	.158	7	.200*	.922	7	.482
	NR+Cangrelor	.350	8	.005	.698	8	.002

*. This is a lower bound of the true significance.

a. Lilliefors Significance Correction

Step2: According to the step1 results, AAR and IS ues t-test, LDH use Mann-Whitney U test:

Independent Samples Test

		Levene's Test for Equality of Variances F	Sig.	t	df	Sig. (2-tailed)
AAR_S3C	Equal variances assumed	.940	.350	-.921	13	.374
	Equal variances not assumed			-.953	11.696	.360
IS_S3C	Equal variances assumed	7.949	.014	2.849	13	.014
	Equal variances not assumed			3.036	7.920	.016

Hypothesis Test Summary

	Null Hypothesis	Test	Sig.	Decision
1	The distribution of LDH_Cangrelor is the same across categories of group_Cangrelor.	Independent-Samples Mann-Whitney U Test	.029[a]	Reject the null hypothesis.

Asymptotic significances are displayed. The significance level is .050.

a. Exact significance is displayed for this test.

Figure 5e and f:

We used two-way Repeated Measures ANOVA applying the Bonferroni multiple comparisons test,

By doing this we first perform the Mauchly's test of Sphericity, then we go further with tests of within-subjects effects and Pairwise comparisons, see the results below:

MAP results

Mauchly's Test of Sphericity[a]

Measure: MEASURE_1

Within Subjects Effect	Mauchly's W	Approx. Chi-Square	df	Sig.	Epsilon[b] Greenhouse-Geisser	Huynh-Feldt	Lower-bound
time	.000	93.065	54	.002	.402	.650	.100

Tests the null hypothesis that the error covariance matrix of the orthonormalized transformed dependent variables is proportional to an identity matrix.

a. Design: Intercept + Group_Congrelor
Within Subjects Design: time

b. May be used to adjust the degrees of freedom for the averaged tests of significance. Corrected tests are displayed in the Tests of Within-Subjects Effects table.

Tests of Within-Subjects Effects

Measure: MEASURE_1

Source		Type IV Sum of Squares	df	Mean Square	F	Sig.
time	Sphericity Assumed	9894.546	10	989.455	7.759	.000
	Greenhouse-Geisser	9894.546	4.023	2459.452	7.759	.000
	Huynh-Feldt	9894.546	6.500	1522.343	7.759	.000
	Lower-bound	9894.546	1.000	9894.546	7.759	.015
time * Group_Congrelor	Sphericity Assumed	2229.289	10	222.929	1.748	.077
	Greenhouse-Geisser	2229.289	4.023	554.127	1.748	.153
	Huynh-Feldt	2229.289	6.500	342.991	1.748	.114
	Lower-bound	2229.289	1.000	2229.289	1.748	.209
Error(time)	Sphericity Assumed	16577.693	130	127.521		
	Greenhouse-Geisser	16577.693	52.300	316.974		
	Huynh-Feldt	16577.693	84.494	196.199		
	Lower-bound	16577.693	13.000	1275.207		

Pairwise Comparisons

Measure: MEASURE_1

(I) time	(J) time	Mean Difference (I-J)	Std. Error	Sig.[b]	95% Confidence Interval for Difference[b]	
					Lower Bound	Upper Bound
1	2	-22.093*	3.911	.004	-38.800	-5.387
	3	-4.354	3.421	1.000	-18.967	10.259
	4	4.467	3.782	1.000	-11.690	20.625
	5	-.415	2.350	1.000	-10.453	9.623
	6	-4.487	3.762	1.000	-20.560	11.587
	7	8.170	4.656	1.000	-11.722	28.061
	8	2.903	4.157	1.000	-14.854	20.661
	9	4.522	3.850	1.000	-11.928	20.972
	10	4.358	4.235	1.000	-13.734	22.449
	11	1.755	3.857	1.000	-14.725	18.234
2	1	22.093*	3.911	.004	5.387	38.800
	3	17.739	4.773	.142	-2.652	38.131
	4	26.561*	6.087	.042	.556	52.566
	5	21.678*	4.984	.043	.384	42.972
	6	17.607	5.744	.497	-6.931	42.144
	7	30.263*	6.092	.014	4.237	56.289
	8	24.996*	5.243	.020	2.598	47.395
	9	26.615*	4.081	.001	9.179	44.051
	10	26.451*	4.921	.007	5.427	47.475
	11	23.848*	3.151	.000	10.386	37.311
3	1	4.354	3.421	1.000	-10.259	18.967
	2	-17.739	4.773	.142	-38.131	2.652
	4	8.821	3.824	1.000	-7.515	25.158
	5	3.938	3.548	1.000	-11.220	19.097
	6	-.133	4.350	1.000	-18.715	18.450
	7	12.524	4.439	.793	-6.439	31.486
	8	7.257	4.177	1.000	-10.589	25.103
	9	8.876	3.896	1.000	-7.767	25.519
	10	8.711	4.409	1.000	-10.126	27.549
	11	6.109	3.836	1.000	-10.280	22.498
4	1	-4.467	3.782	1.000	-20.625	11.690
	2	-26.561*	6.087	.042	-52.566	-.556
	3	-8.821	3.824	1.000	-25.158	7.515
	5	-4.883	3.980	1.000	-21.885	12.119
	6	-8.954	3.987	1.000	-25.986	8.078
	7	3.702	4.008	1.000	-13.421	20.825
	8	-1.564	4.615	1.000	-21.281	18.152
	9	.054	5.079	1.000	-21.644	21.753
	10	-.110	4.868	1.000	-20.907	20.687
	11	-2.713	5.882	1.000	-27.843	22.418
5	1	.415	2.350	1.000	-9.623	10.453
	2	-21.678*	4.984	.043	-42.972	-.384
	3	-3.938	3.548	1.000	-19.097	11.220
	4	4.883	3.980	1.000	-12.119	21.885
	6	-4.071	3.214	1.000	-17.801	9.658
	7	8.585	4.923	1.000	-12.448	29.618
	8	3.319	4.367	1.000	-15.339	21.977
	9	4.937	4.370	1.000	-13.731	23.606
	10	4.773	4.084	1.000	-12.673	22.219
	11	2.170	4.254	1.000	-16.004	20.344
6	1	4.487	3.762	1.000	-11.587	20.560
	2	-17.607	5.744	.497	-42.144	6.931
	3	.133	4.350	1.000	-18.450	18.715
	4	8.954	3.987	1.000	-8.078	25.986
	5	4.071	3.214	1.000	-9.658	17.801
	7	12.656	3.388	.137	-1.819	27.132
	8	7.390	3.452	1.000	-7.359	22.139
	9	9.009	3.568	1.000	-6.234	24.251
	10	8.844	3.766	1.000	-7.245	24.934
	11	6.242	4.653	1.000	-13.638	26.121
7	1	-8.170	4.656	1.000	-28.061	11.722
	2	-30.263*	6.092	.014	-56.289	-4.237
	3	-12.524	4.439	.793	-31.486	6.439
	4	-3.702	4.008	1.000	-20.825	13.421
	5	-8.585	4.923	1.000	-29.618	12.448
	6	-12.656	3.388	.137	-27.132	1.819
	8	-5.267	1.982	1.000	-13.733	3.200
	9	-3.648	2.732	1.000	-15.321	8.026
	10	-3.812	2.950	1.000	-16.414	8.790
	11	-6.415	4.894	1.000	-27.324	14.494
8	1	-2.903	4.157	1.000	-20.661	14.854
	2	-24.996*	5.243	.020	-47.395	-2.598
	3	-7.257	4.177	1.000	-25.103	10.589
	4	1.564	4.615	1.000	-18.152	21.281
	5	-3.319	4.367	1.000	-21.977	15.339
	6	-7.390	3.452	1.000	-22.139	7.359
	7	5.267	1.982	1.000	-3.200	13.733
	9	1.619	1.946	1.000	-6.697	9.934
	10	1.454	1.824	1.000	-6.336	9.245
	11	-1.148	4.034	1.000	-18.384	16.087
9	1	-4.522	3.850	1.000	-20.972	11.928
	2	-26.615*	4.081	.001	-44.051	-9.179
	3	-8.876	3.896	1.000	-25.519	7.767
	4	-.054	5.079	1.000	-21.753	21.644
	5	-4.937	4.370	1.000	-23.606	13.731
	6	-9.009	3.568	1.000	-24.251	6.234
	7	3.648	2.732	1.000	-8.026	15.321
	8	-1.619	1.946	1.000	-9.934	6.697
	10	-.164	2.350	1.000	-10.202	9.874
	11	-2.767	2.701	1.000	-14.306	8.772
10	1	-4.358	4.235	1.000	-22.449	13.734
	2	-26.451*	4.921	.007	-47.475	-5.427
	3	-8.711	4.409	1.000	-27.549	10.126
	4	.110	4.868	1.000	-20.687	20.907
	5	-4.773	4.084	1.000	-22.219	12.673
	6	-8.844	3.766	1.000	-24.934	7.245
	7	3.812	2.950	1.000	-8.790	16.414
	8	-1.454	1.824	1.000	-9.245	6.336
	9	.164	2.350	1.000	-9.874	10.202
	11	-2.603	3.623	1.000	-18.081	12.875
11	1	-1.755	3.857	1.000	-18.234	14.725
	2	-23.848*	3.151	.000	-37.311	-10.386
	3	-6.109	3.836	1.000	-22.498	10.280
	4	2.713	5.882	1.000	-22.418	27.843
	5	-2.170	4.254	1.000	-20.344	16.004
	6	-6.242	4.653	1.000	-26.121	13.638
	7	6.415	4.894	1.000	-14.494	27.324
	8	1.148	4.034	1.000	-16.087	18.384
	9	2.767	2.701	1.000	-8.772	14.306
	10	2.603	3.623	1.000	-12.875	18.081

Based on estimated marginal means

*. The mean difference is significant at the .05 level.

b. Adjustment for multiple comparisons: Bonferroni.

Tests of Between-Subjects Effects

Measure: MEASURE_1

Transformed Variable: Average

Source	Type IV Sum of Squares	df	Mean Square	F	Sig.
Intercept	782989.820	1	782989.820	328.069	.000
Group_Congrelor	4986.006	1	4986.006	2.089	.172
Error	31026.625	13	2386.663		

Glucose results

Mauchly's Test of Sphericity[a]

Measure: MEASURE_1

Within Subjects Effect	Mauchly's W	Approx. Chi-Square	df	Sig.	Epsilon[b] Greenhouse-Geisser	Huynh-Feldt	Lower-bound
time	.636	5.432	2	.066	.733	.867	.500

Tests the null hypothesis that the error covariance matrix of the orthonormalized transformed dependent variables is proportional to an identity matrix.

a. Design: Intercept + Group_Congrelor
Within Subjects Design: time

b. May be used to adjust the degrees of freedom for the averaged tests of significance. Corrected tests are displayed in the Tests of Within-Subjects Effects table.

Tests of Within-Subjects Effects

Measure: MEASURE_1

Source		Type IV Sum of Squares	df	Mean Square	F	Sig.
time	Sphericity Assumed	2.821	2	1.411	1.360	.274
	Greenhouse-Geisser	2.821	1.466	1.924	1.360	.273
	Huynh-Feldt	2.821	1.733	1.628	1.360	.274
	Lower-bound	2.821	1.000	2.821	1.360	.264
time * Group_Congrelor	Sphericity Assumed	7.980	2	3.990	3.847	.034
	Greenhouse-Geisser	7.980	1.466	5.442	3.847	.051
	Huynh-Feldt	7.980	1.733	4.603	3.847	.042
	Lower-bound	7.980	1.000	7.980	3.847	.072
Error(time)	Sphericity Assumed	26.967	26	1.037		
	Greenhouse-Geisser	26.967	19.061	1.415		
	Huynh-Feldt	26.967	22.534	1.197		
	Lower-bound	26.967	13.000	2.074		

Tests of Between-Subjects Effects

Measure: MEASURE_1

Transformed Variable: Average

Source	Type IV Sum of Squares	df	Mean Square	F	Sig.
Intercept	2364.670	1	2364.670	1297.934	.000
Group_Congrelor	.552	1	.552	.303	.591
Error	23.684	13	1.822		

Figure6a-d.

Step 1: Test of normality:

Tests of Normality

	Group_S3_prop	Kolmogorov-Smirnov[a] Statistic	df	Sig.	Shapiro-Wilk Statistic	df	Sig.
LV_S3P	Saline+propofol	.196	5	.200*	.979	5	.928
	NR+propofol	.262	5	.200*	.919	5	.525
AAR_S3P	Saline+propofol	.261	5	.200*	.876	5	.293
	NR+propofol	.132	5	.200*	.997	5	.997
IS_S3P	Saline+propofol	.263	5	.200*	.845	5	.180
	NR+propofol	.163	5	.200*	.993	5	.989

Tests of Normality

	group_Propofol	Kolmogorov-Smirnov[a] Statistic	df	Sig.	Shapiro-Wilk Statistic	df	Sig.
LDH_Propofol	Saline+propofol	.252	6	.200*	.899	6	.369
	NR+propofol	.219	6	.200*	.952	6	.753

*. This is a lower bound of the true significance.

a. Lilliefors Significance Correction

Step 2: use independent t-test according to normality test results above:

Independent Samples Test

		Levene's Test for Equality of Variances		t-test for Equality of Means						
									95% Confidence Interval of the Difference	
		F	Sig.	t	df	Sig. (2-tailed)	Mean Difference	Std. Error Difference	Lower	Upper
LV_S3P	Equal variances assumed	.142	.714	.436	10	.672	25286.16667	58055.65521	-104069.894	154642.2276
	Equal variances not assumed			.436	9.631	.673	25286.16667	58055.65521	-104744.673	155317.0067
AAR_S3P	Equal variances assumed	2.047	.183	1.841	10	.095	6.45735	3.50775	-1.35840	14.27310
	Equal variances not assumed			1.841	8.141	.102	6.45735	3.50775	-1.60728	14.52199
IS_S3P	Equal variances assumed	.838	.382	-.209	10	.839	-1.79859	8.61683	-20.99809	17.40091
	Equal variances not assumed			-.209	9.862	.839	-1.79859	8.61683	-21.03454	17.43737

Independent Samples Test

		Levene's Test for Equality of Variances		t-test for Equality of Means						
									95% Confidence Interval of the Difference	
		F	Sig.	t	df	Sig. (2-tailed)	Mean Difference	Std. Error Difference	Lower	Upper
LDH_Propofol	Equal variances assumed	.021	.887	-.002	10	.999	-.00007	.03925	-.08752	.08738
	Equal variances not assumed			-.002	9.880	.999	-.00007	.03925	-.08767	.08752

Figure 6e and f:

We used two-way Repeated Measures ANOVA applying the Bonferroni multiple comparisons test,

By doing this we first perform the Mauchly's test of Sphericity, then we go further with tests of within-subjects effects and Pairwise comparisons, see the results below:

MAP results:

Mauchly's Test of Sphericity[a]

Measure: MEASURE_1

Within Subjects Effect	Mauchly's W	Approx. Chi-Square	df	Sig.	Epsilon[b] Greenhouse-Geisser	Epsilon[b] Huynh-Feldt	Epsilon[b] Lower-bound
time	.000	84.709	54	.020	.479	1.000	.100

Tests the null hypothesis that the error covariance matrix of the orthonormalized transformed dependent variables is proportional to an identity matrix.

a. Design: Intercept + Group_Propofol
Within Subjects Design: time

b. May be used to adjust the degrees of freedom for the averaged tests of significance. Corrected tests are displayed in the Tests of Within-Subjects Effects table.

Tests of Within-Subjects Effects

Measure: MEASURE_1

Source		Type III Sum of Squares	df	Mean Square	F	Sig.
time	Sphericity Assumed	23799.620	10	2379.962	3.108	.002
	Greenhouse-Geisser	23799.620	4.795	4963.561	3.108	.018
	Huynh-Feldt	23799.620	10.000	2379.962	3.108	.002
	Lower-bound	23799.620	1.000	23799.620	3.108	.108
time * Group_Propofol	Sphericity Assumed	8454.262	10	845.426	1.104	.367
	Greenhouse-Geisser	8454.262	4.795	1763.190	1.104	.370
	Huynh-Feldt	8454.262	10.000	845.426	1.104	.367
	Lower-bound	8454.262	1.000	8454.262	1.104	.318
Error(time)	Sphericity Assumed	76584.867	100	765.849		
	Greenhouse-Geisser	76584.867	47.949	1597.226		
	Huynh-Feldt	76584.867	100.000	765.849		
	Lower-bound	76584.867	10.000	7658.487		

Pairwise Comparisons

Measure: MEASURE_1

(I) time	(J) time	Mean Difference (I-J)	Std. Error	Sig.[b]	95% Confidence Interval for Difference[b] Lower Bound	95% Confidence Interval for Difference[b] Upper Bound
1	2	-20.372	7.877	1.000	-56.993	16.248
	3	-23.800	15.094	1.000	-93.977	46.377
	4	-7.682	11.185	1.000	-59.684	44.320
	5	-9.758	15.391	1.000	-81.315	61.798
	6	-10.852	14.086	1.000	-76.342	54.637
	7	14.532	11.287	1.000	-37.945	67.008
	8	-11.549	13.407	1.000	-73.880	50.782
	9	8.189	12.853	1.000	-51.569	67.947
	10	10.033	10.328	1.000	-37.985	58.052
	11	17.859	10.972	1.000	-33.154	68.873
2	1	20.372	7.877	1.000	-16.248	56.993
	3	-3.428	12.641	1.000	-62.198	55.343
	4	12.691	11.029	1.000	-38.585	63.967
	5	10.614	15.147	1.000	-59.806	81.034
	6	9.520	12.047	1.000	-46.491	65.531
	7	34.904	9.591	.250	-9.686	79.494
	8	8.823	11.105	1.000	-42.805	60.451
	9	28.562	14.103	1.000	-37.008	94.131
	10	30.406	8.981	.382	-11.349	72.161
	11	38.232	9.578	.140	-6.298	82.762
3	1	23.800	15.094	1.000	-46.377	93.977
	2	3.428	12.641	1.000	-55.343	62.198
	4	16.118	9.495	1.000	-28.027	60.264
	5	14.042	12.385	1.000	-43.539	71.622
	6	12.948	14.031	1.000	-52.285	78.180
	7	38.332	11.252	.368	-13.981	90.644
	8	12.251	12.317	1.000	-45.014	69.515
	9	31.989	15.845	1.000	-41.678	105.657
	10	33.833	14.676	1.000	-34.399	102.065
	11	41.659	10.989	.195	-9.433	92.751
4	1	7.682	11.185	1.000	-44.320	59.684
	2	-12.691	11.029	1.000	-63.967	38.585
	3	-16.118	9.495	1.000	-60.264	28.027
	5	-2.077	11.251	1.000	-54.384	50.231
	6	-3.171	9.877	1.000	-49.091	42.750
	7	22.213	7.537	.803	-12.830	57.256
	8	-3.868	13.353	1.000	-65.951	58.216
	9	15.871	11.822	1.000	-39.092	70.834
	10	17.715	12.099	1.000	-38.538	73.968
	11	25.541	10.321	1.000	-22.446	73.528
5	1	9.758	15.391	1.000	-61.798	81.315
	2	-10.614	15.147	1.000	-81.034	59.806
	3	-14.042	12.385	1.000	-71.622	43.539
	4	2.077	11.251	1.000	-50.231	54.384
	6	-1.094	10.359	1.000	-49.254	47.066
	7	24.290	12.049	1.000	-31.727	80.307
	8	-1.791	11.420	1.000	-54.887	51.305
	9	17.948	9.943	1.000	-28.278	64.173
	10	19.792	12.421	1.000	-37.956	77.539
	11	27.617	8.446	.464	-11.648	66.883
6	1	10.852	14.086	1.000	-54.637	76.342
	2	-9.520	12.047	1.000	-65.531	46.491
	3	-12.948	14.031	1.000	-78.180	52.285
	4	3.171	9.877	1.000	-42.750	49.091
	5	1.094	10.359	1.000	-47.066	49.254
	7	25.384	7.549	.397	-9.714	60.482
	8	-.697	12.537	1.000	-58.983	57.590
	9	19.042	10.301	1.000	-28.851	66.935
	10	20.886	8.818	1.000	-20.111	61.883
	11	28.712	10.023	.925	-17.887	75.310
7	1	-14.532	11.287	1.000	-67.008	37.945
	2	-34.904	9.591	.250	-79.494	9.686
	3	-38.332	11.252	.368	-90.644	13.981
	4	-22.213	7.537	.803	-57.256	12.830
	5	-24.290	12.049	1.000	-80.307	31.727
	6	-25.384	7.549	.397	-60.482	9.714
	8	-26.081	12.726	1.000	-85.245	33.083
	9	-6.342	9.987	1.000	-52.773	40.088
	10	-4.498	9.365	1.000	-48.038	39.042
	11	3.328	9.155	1.000	-39.235	45.890
8	1	11.549	13.407	1.000	-50.782	73.880
	2	-8.823	11.105	1.000	-60.451	42.805
	3	-12.251	12.317	1.000	-69.515	45.014
	4	3.868	13.353	1.000	-58.216	65.951
	5	1.791	11.420	1.000	-51.305	54.887
	6	.697	12.537	1.000	-57.590	58.983
	7	26.081	12.726	1.000	-33.083	85.245
	9	19.738	10.240	1.000	-27.868	67.344
	10	21.583	9.067	1.000	-20.570	63.735
	11	29.408*	5.850	.028	2.208	56.608
9	1	-8.189	12.853	1.000	-67.947	51.569
	2	-28.562	14.103	1.000	-94.131	37.008
	3	-31.989	15.845	1.000	-105.657	41.678
	4	-15.871	11.822	1.000	-70.834	39.092
	5	-17.948	9.943	1.000	-64.173	28.278
	6	-19.042	10.301	1.000	-66.935	28.851
	7	6.342	9.987	1.000	-40.088	52.773
	8	-19.738	10.240	1.000	-67.344	27.868
	10	1.844	9.569	1.000	-42.645	46.334
	11	9.670	8.240	1.000	-28.638	47.978
10	1	-10.033	10.328	1.000	-58.052	37.985
	2	-30.406	8.981	.382	-72.161	11.349
	3	-33.833	14.676	1.000	-102.065	34.399
	4	-17.715	12.099	1.000	-73.968	38.538
	5	-19.792	12.421	1.000	-77.539	37.956
	6	-20.886	8.818	1.000	-61.883	20.111
	7	4.498	9.365	1.000	-39.042	48.038
	8	-21.583	9.067	1.000	-63.735	20.570
	9	-1.844	9.569	1.000	-46.334	42.645
	11	7.826	7.096	1.000	-25.167	40.819
11	1	-17.859	10.972	1.000	-68.873	33.154
	2	-38.232	9.578	.140	-82.762	6.298
	3	-41.659	10.989	.195	-92.751	9.433
	4	-25.541	10.321	1.000	-73.528	22.446
	5	-27.617	8.446	.464	-66.883	11.648
	6	-28.712	10.023	.925	-75.310	17.887
	7	-3.328	9.155	1.000	-45.890	39.235
	8	-29.408*	5.850	.028	-56.608	-2.208
	9	-9.670	8.240	1.000	-47.978	28.638
	10	-7.826	7.096	1.000	-40.819	25.167

[illegible]	[illegible]	[illegible]	[illegible]	[illegible]	[illegible]	[illegible]
	2	-9.520	12.047	1.000	-65.531	46.491
	3	-12.948	14.031	1.000	-78.180	52.285
	4	3.171	9.877	1.000	-42.750	49.091
	5	1.094	10.359	1.000	-47.066	49.254
	7	25.384	7.549	.397	-9.714	60.482
	8	-.697	12.537	1.000	-58.983	57.590
	9	19.042	10.301	1.000	-28.851	66.935
	10	20.886	8.818	1.000	-20.111	61.883
	11	28.712	10.023	.925	-17.887	75.310

Based on estimated marginal means

*. The mean difference is significant at the .05 level.

b. Adjustment for multiple comparisons: Bonferroni.

Glucose results:

Tests of Within-Subjects Effects

Measure: MEASURE_1

Source		Type III Sum of Squares	df	Mean Square	F	Sig.
time	Sphericity Assumed	23.502	2	11.751	11.478	.000
	Greenhouse-Geisser	23.502	1.774	13.250	11.478	.001
	Huynh-Feldt	23.502	2.000	11.751	11.478	.000
	Lower-bound	23.502	1.000	23.502	11.478	.007
time * Group_Propofol	Sphericity Assumed	.809	2	.404	.395	.679
	Greenhouse-Geisser	.809	1.774	.456	.395	.655
	Huynh-Feldt	.809	2.000	.404	.395	.679
	Lower-bound	.809	1.000	.809	.395	.544
Error(time)	Sphericity Assumed	20.476	20	1.024		
	Greenhouse-Geisser	20.476	17.737	1.154		
	Huynh-Feldt	20.476	20.000	1.024		
	Lower-bound	20.476	10.000	2.048		

Pairwise Comparisons

Measure: MEASURE_1

(I) time	(J) time	Mean Difference (I-J)	Std. Error	Sig.[b]	95% Confidence Interval for Difference[b]	
					Lower Bound	Upper Bound
1	2	-1.333*	.450	.043	-2.626	-.040
	3	.600	.331	.301	-.351	1.551
2	1	1.333*	.450	.043	.040	2.626
	3	1.933*	.446	.004	.652	3.214
3	1	-.600	.331	.301	-1.551	.351
	2	-1.933*	.446	.004	-3.214	-.652

Based on estimated marginal means

*. The mean difference is significant at the .05 level.

b. Adjustment for multiple comparisons: Bonferroni.

Table1:

test of normality and following comparison:

Series1:

Tests of Normality

	Group_S2_NR+Mel	Kolmogorov-Smirnov[a] Statistic	df	Sig.	Shapiro-Wilk Statistic	df	Sig.
BW	Saline	.343	6	.027	.772	6	.032
	Saline + Ethanol	.151	6	.200*	.988	6	.985
	Melatonin	.279	7	.107	.877	7	.213
	NR	.378	8	.001	.721	8	.004
	NR+Melatonin	.202	7	.200*	.962	7	.839
HR_initial	Saline	.156	6	.200*	.968	6	.877
	Saline + Ethanol	.214	6	.200*	.968	6	.878
	Melatonin	.260	7	.168	.903	7	.351
	NR	.124	8	.200*	.946	8	.667
	NR+Melatonin	.180	7	.200*	.933	7	.579
MAP_initial	Saline	.231	6	.200*	.904	6	.400
	Saline + Ethanol	.165	6	.200*	.979	6	.946
	Melatonin	.322	7	.027	.855	7	.138
	NR	.226	8	.200*	.854	8	.106
	NR+Melatonin	.176	7	.200*	.902	7	.346

BW:

Independent-Samples Kruskal-Wallis Test Summary

Total N	35
Test Statistic	5.364[a,b]
Degree Of Freedom	4
Asymptotic Sig.(2-sided test)	.252

a. The test statistic is adjusted for ties.

b. Multiple comparisons are not performed because the overall test does not show significant differences across samples.

Test of Homogeneity of Variances

		Levene Statistic	df1	df2	Sig.
HR_initial	Based on Mean	.365	4	29	.832
	Based on Median	.236	4	29	.916
	Based on Median and with adjusted df	.236	4	26.559	.916
	Based on trimmed mean	.360	4	29	.835
MAP_initial	Based on Mean	1.075	4	29	.387
	Based on Median	.926	4	29	.462
	Based on Median and with adjusted df	.926	4	28.034	.463
	Based on trimmed mean	1.087	4	29	.381

ANOVA

				Sum of Squares	df	Mean Square	F	Sig.
HR_initial	Between Groups	(Combined)		2834.620	4	708.655	.881	.487
		Linear Term	Unweighted	847.802	1	847.802	1.054	.313
			Weighted	913.302	1	913.302	1.135	.295
			Deviation	1921.318	3	640.439	.796	.506
	Within Groups			23327.457	29	804.395		
	Total			26162.076	33			
MAP_initial	Between Groups	(Combined)		672.255	4	168.064	1.759	.164
		Linear Term	Unweighted	90.269	1	90.269	.945	.339
			Weighted	82.624	1	82.624	.865	.360
			Deviation	589.631	3	196.544	2.057	.128
	Within Groups			2771.510	29	95.569		
	Total			3443.765	33			

Series2 and 3:

Tests of Normality

	GROUP	Kolmogorov-Smirnov[a] Statistic	df	Sig.	Shapiro-Wilk Statistic	df	Sig.
BW	Saline+ cangrelor	.236	7	.200*	.885	7	.249
	NR+ cangrelor	.300	8	.033	.905	8	.320
	Saline+ Propofol	.174	6	.200*	.953	6	.762
	NR+ Propofol	.266	6	.200*	.839	6	.129
HR	Saline+ cangrelor	.196	7	.200*	.934	7	.585
	NR+ cangrelor	.167	8	.200*	.960	8	.808
	Saline+ Propofol	.150	6	.200*	.972	6	.904
	NR+ Propofol	.223	6	.200*	.868	6	.220
MAP	Saline+ cangrelor	.258	7	.176	.820	7	.064
	NR+ cangrelor	.298	8	.035	.853	8	.101
	Saline+ Propofol	.243	6	.200*	.920	6	.505
	NR+ Propofol	.273	6	.183	.837	6	.124

*. This is a lower bound of the true significance.

a. Lilliefors Significance Correction

Independent Samples Test

		Levene's Test for Equality of Variances		t-test for Equality of Means						
									95% Confidence Interval of the Difference	
		F	Sig.	t	df	Sig. (2-tailed)	Mean Difference	Std. Error Difference	Lower	Upper
BW	Equal variances assumed	.137	.718	1.231	13	.240	16.30357	13.24686	-12.31452	44.92166
	Equal variances not assumed			1.227	12.542	.243	16.30357	13.29193	-12.51887	45.12602
HR	Equal variances assumed	.038	.848	.288	13	.778	5.69107	19.78420	-37.05009	48.43224
	Equal variances not assumed			.289	12.960	.777	5.69107	19.65951	-36.79403	48.17617
MAP	Equal variances assumed	.112	.743	-.265	13	.795	-2.40000	9.06840	-21.99109	17.19109
	Equal variances not assumed			-.262	12.125	.798	-2.40000	9.15072	-22.31484	17.51484

Independent Samples Test

		Levene's Test for Equality of Variances		t-test for Equality of Means						
									95% Confidence Interval of the Difference	
		F	Sig.	t	df	Sig. (2-tailed)	Mean Difference	Std. Error Difference	Lower	Upper
BW	Equal variances assumed	1.237	.292	1.217	10	.252	17.33333	14.24508	-14.40668	49.07334
	Equal variances not assumed			1.217	8.590	.256	17.33333	14.24508	-15.12702	49.79369
HR	Equal variances assumed	.714	.418	1.660	10	.128	38.20000	23.01169	-13.07324	89.47324
	Equal variances not assumed			1.660	8.963	.131	38.20000	23.01169	-13.88909	90.28909
MAP	Equal variances assumed	.298	.597	.513	10	.619	11.42833	22.26261	-38.17584	61.03251
	Equal variances not assumed			.513	9.997	.619	11.42833	22.26261	-38.17816	61.03483

Table2:

MAP:

Multivariate Tests[a]

Effect		Value	F	Hypothesis df	Error df	Sig.
time	Pillai's Trace	.475	5.657[b]	4.000	25.000	.002
	Wilks' Lambda	.525	5.657[b]	4.000	25.000	.002
	Hotelling's Trace	.905	5.657[b]	4.000	25.000	.002
	Roy's Largest Root	.905	5.657[b]	4.000	25.000	.002
time * Group	Pillai's Trace	.362	.696	16.000	112.000	.793
	Wilks' Lambda	.670	.674	16.000	77.014	.811
	Hotelling's Trace	.445	.654	16.000	94.000	.831
	Roy's Largest Root	.291	2.038[c]	4.000	28.000	.116

a. Design: Intercept + Group
Within Subjects Design: time

b. Exact statistic

c. The statistic is an upper bound on F that yields a lower bound on the significance level.

Mauchly's Test of Sphericity[a]

Measure: MEASURE_1

Within Subjects Effect	Mauchly's W	Approx. Chi-Square	df	Sig.	Epsilon[b] Greenhouse-Geisser	Huynh-Feldt	Lower-bound
time	.628	12.299	9	.198	.809	1.000	.250

Tests the null hypothesis that the error covariance matrix of the orthonormalized transformed dependent variables is proportional to an identity matrix.

a. Design: Intercept + Group
Within Subjects Design: time

b. May be used to adjust the degrees of freedom for the averaged tests of significance. Corrected tests are displayed in the Tests of Within-Subjects Effects table.

Tests of Within-Subjects Effects

Measure: MEASURE_1

Source		Type IV Sum of Squares	df	Mean Square	F	Sig.
time	Sphericity Assumed	1879.891	4	469.973	5.580	.000
	Greenhouse-Geisser	1879.891	3.236	580.861	5.580	.001
	Huynh-Feldt	1879.891	4.000	469.973	5.580	.000
	Lower-bound	1879.891	1.000	1879.891	5.580	.025
time * Group	Sphericity Assumed	1227.612	16	76.726	.911	.559
	Greenhouse-Geisser	1227.612	12.946	94.829	.911	.545
	Huynh-Feldt	1227.612	16.000	76.726	.911	.559
	Lower-bound	1227.612	4.000	306.903	.911	.471
Error(time)	Sphericity Assumed	9433.458	112	84.227		
	Greenhouse-Geisser	9433.458	90.619	104.100		
	Huynh-Feldt	9433.458	112.000	84.227		
	Lower-bound	9433.458	28.000	336.909		

Pairwise Comparisons

Measure: MEASURE_1

(I) time	(J) time	Mean Difference (I-J)	Std. Error	Sig.[b]	95% Confidence Interval for Difference[b] Lower Bound	Upper Bound
1	2	8.786*	2.102	.003	2.382	15.190
	3	-.385	1.686	1.000	-5.524	4.753
	4	2.229	2.378	1.000	-5.017	9.476
	5	.437	2.708	1.000	-7.814	8.688
2	1	-8.786*	2.102	.003	-15.190	-2.382
	3	-9.171*	1.914	.000	0.000490 [illegible]003	-3.340
	4	-6.557	2.381	.102	-13.813	.699
	5	-8.349	2.804	.059	-16.894	.196
3	1	.385	1.686	1.000	-4.753	5.524
	2	9.171*	1.914	.000	3.340	15.003
	4	2.615	1.924	1.000	-3.247	8.476
	5	.822	2.400	1.000	-6.489	8.134
4	1	-2.229	2.378	1.000	-9.476	5.017
	2	6.557	2.381	.102	-.699	13.813
	3	-2.615	1.924	1.000	-8.476	3.247
	5	-1.792	2.252	1.000	-8.654	5.070
5	1	-.437	2.708	1.000	-8.688	7.814
	2	8.349	2.804	.059	-.196	16.894
	3	-.822	2.400	1.000	-8.134	6.489
	4	1.792	2.252	1.000	-5.070	8.654

Based on estimated marginal means

*. The mean difference is significant at the .05 level.

b. Adjustment for multiple comparisons: Bonferroni.

Pairwise Comparisons

Measure: MEASURE_1

(I) Group_MAP	(J) Group_MAP	Mean Difference (I-J)	Std. Error	Sig.[a]	95% Confidence Interval for Difference[a] Lower Bound	Upper Bound
Sailne	saline+Ethanol	-.084	7.422	1.000	-22.699	22.530
	Melatoniin	-3.291	6.775	1.000	-23.936	17.353
	NR	2.717	6.775	1.000	-17.927	23.362
	NR+Melatonin	-5.496	6.775	1.000	-26.140	15.148
saline+Ethanol	Sailne	.084	7.422	1.000	-22.530	22.699
	Melatoniin	-3.207	7.422	1.000	-25.822	19.408
	NR	2.802	7.422	1.000	-19.813	25.417
	NR+Melatonin	-5.412	7.422	1.000	-28.026	17.203
Melatoniin	Sailne	3.291	6.775	1.000	-17.353	23.936
	saline+Ethanol	3.207	7.422	1.000	-19.408	25.822
	NR	6.009	6.775	1.000	-14.636	26.653
	NR+Melatonin	-2.205	6.775	1.000	-22.849	18.440
NR	Sailne	-2.717	6.775	1.000	-23.362	17.927
	saline+Ethanol	-2.802	7.422	1.000	-25.417	19.813
	Melatoniin	-6.009	6.775	1.000	-26.653	14.636
	NR+Melatonin	-8.213	6.775	1.000	-28.858	12.431
NR+Melatonin	Sailne	5.496	6.775	1.000	-15.148	26.140
	saline+Ethanol	5.412	7.422	1.000	-17.203	28.026
	Melatoniin	2.205	6.775	1.000	-18.440	22.849
	NR	8.213	6.775	1.000	-12.431	28.858

Based on estimated marginal means

a. Adjustment for multiple comparisons: Bonferroni.

HR:

Tests of Within-Subjects Effects

Measure: MEASURE_1

Source		Type IV Sum of Squares	df	Mean Square	F	Sig.
time	Sphericity Assumed	12751.317	4	3187.829	2.077	.088
	Greenhouse-Geisser	12751.317	3.239	3937.296	2.077	.103
	Huynh-Feldt	12751.317	4.000	3187.829	2.077	.088
	Lower-bound	12751.317	1.000	12751.317	2.077	.160
time * Group_HR	Sphericity Assumed	11640.924	16	727.558	.474	.955
	Greenhouse-Geisser	11640.924	12.954	898.608	.474	.934
	Huynh-Feldt	11640.924	16.000	727.558	.474	.955
	Lower-bound	11640.924	4.000	2910.231	.474	.754
Error(time)	Sphericity Assumed	184194.048	120	1534.950		
	Greenhouse-Geisser	184194.048	97.158	1895.821		
	Huynh-Feldt	184194.048	120.000	1534.950		
	Lower-bound	184194.048	30.000	6139.802		
