# Supplementary figures and images for: Cardioprotecive Properties of Known Agents in Rat Ischemia-Reperfusion Model Under Clinically Relevant Conditions: Only the NAD Precursor Nicotinamide Riboside Reduces Infarct Size in Presence of Fentanyl, Midazolam and Cangrelor, but Not Propofol
Source: Front Cardiovasc Med. 2021 Aug 30;8:712478. doi: 10.3389/fcvm.2021.712478 (PMC8435675; doi:10.3389/fcvm.2021.712478)

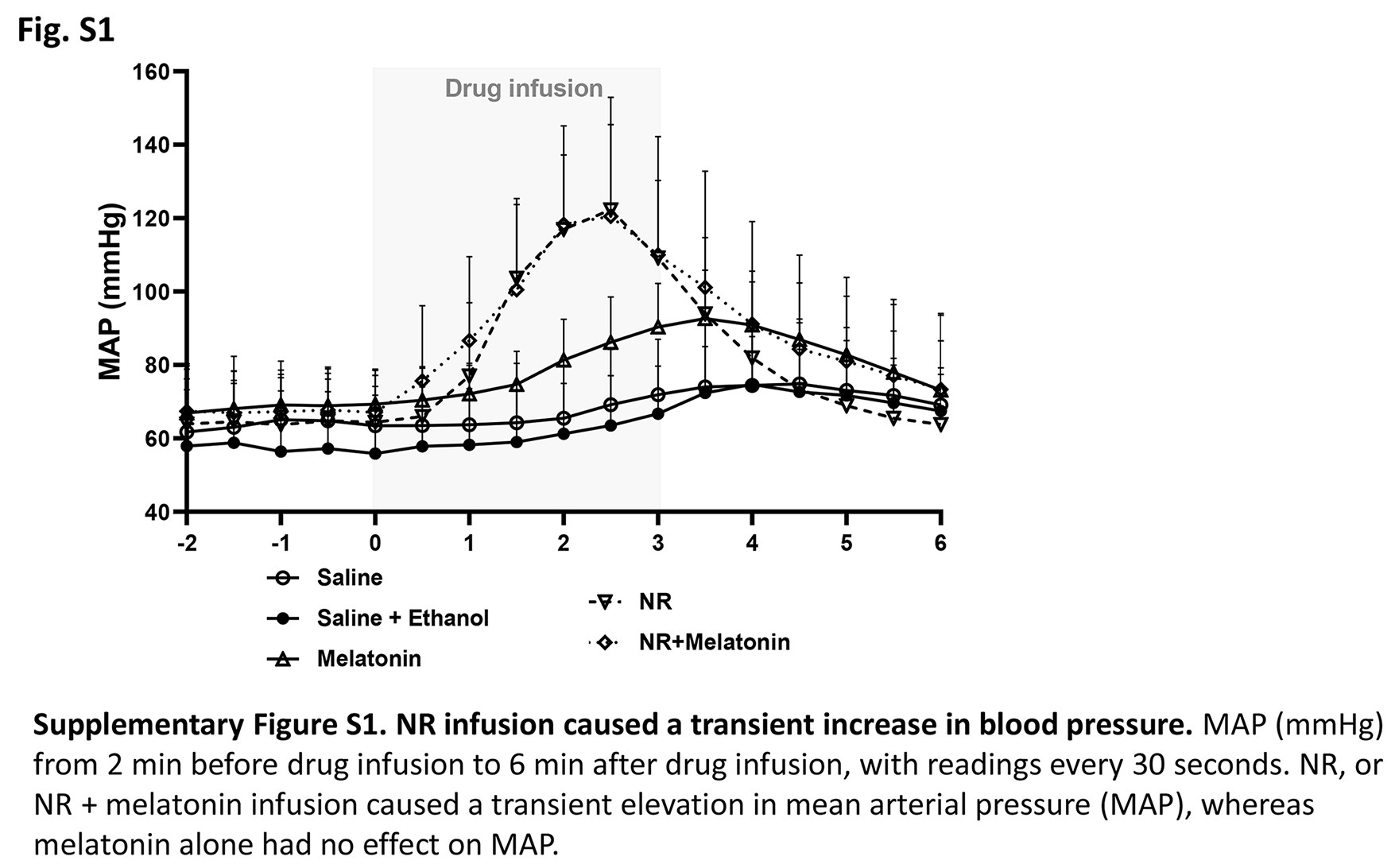

Supplement: Supplementary file 2 [file Image_1.PNG]
